# Omic AI reveals new autophagy regulators from the Atg1 interactome in *Saccharomyces cerevisiae*

**DOI:** 10.3389/fcell.2025.1554958

**Published:** 2025-04-29

**Authors:** Cheng Han, Shanshan Fu, Dachao Tang, Yuting Chen, Dan Liu, Zihao Feng, Yujie Gou, Chi Zhang, Weizhi Zhang, Leming Xiao, Jiayi Zhang, Cong Yi, Yu Xue, Di Peng

**Affiliations:** ^1^ MOE Key Laboratory of Molecular Biophysics, Hubei Bioinformatics and Molecular Imaging Key Laboratory, Center for Artificial Intelligence Biology, College of Life Science and Technology, Huazhong University of Science and Technology, Wuhan, Hubei, China; ^2^ Department of Biochemistry, and Department of Hepatobiliary and Pancreatic Surgery of the First Affiliated Hospital, Zhejiang University School of Medicine, Hangzhou, China; ^3^ Nanjing University Institute of Artificial Intelligence Biomedicine, Nanjing, Jiangsu, China

**Keywords:** Atg1, autophagy-related, protein kinase, artificial intelligence, deep learning, phosphorylation

## Abstract

In *Saccharomyces cerevisiae*, Atg1 is a core autophagy-related (Atg) protein kinase (PK) in regulating macroautophagy/autophagy, by physically interacting with numerous other proteins, or by phosphorylating various substrates. It is unclear how many Atg1-interacting partners and substrates are also involved in regulating autophagy. Here, we conducted transcriptomic, proteomic and phosphoproteomic profiling of Atg1-dependent molecular landscapes during nitrogen starvation-triggered autophagy, and detected 244, 245 and 217 genes to be affected by *ATG1* in the autophagic process at mRNA, protein, and phosphorylation levels, respectively. Based on the Atg1 interactome, we developed a novel artificial intelligence (AI) framework, inference of autophagy regulators from multi-omic data (iAMD), and predicted 12 Atg1-interacting partners and 17 substrates to be potentially functional in autophagy. Further experiments validated that Rgd1 and Whi5 are required for bulk autophagy, as well as physical interactions and co-localizations with Atg1 during autophagy. In particular, we demonstrated that 2 phosphorylation sites (p-sites), pS78 and pS149 of Whi5, are phosphorylated by Atg1 to regulate the formation of Atg1 puncta during autophagy initiation. A working model was illustrated to emphasize the importance of the Atg1-centered network in yeast autophagy. In addition, iAMD was extended to accurately predict Atg proteins and autophagy regulators from other PK interactomes, indicating a high transferability of the method. Taken together, we not only revealed new autophagy regulators from the Atg1 interactome, but also provided a useful resource for further analysis of yeast autophagy.

## 1 Introduction

Macroautophagy (hereafter referred to as autophagy) is a major lysosome-dependent degradative and recycling pathway in eukaryotic cells, playing an essential role in maintaining cellular homeostasis and cell survival (Ohsumi, 2014; Klionsky et al., 2021). The occurrence of autophagy is characterized by formation of double-membrane structures, termed autophagosomes, in response to a variety of extracellular environmental stresses, such as nutrient starvation, chemical reagents and oxidative stress (Klionsky et al., 2021). In cytoplasm, excessive or aberrant proteins and/or organelles are sequestered by autophagosomes and subsequently delivered into vacuoles/lysosomes for degradation (Mizushima, 2010; Hurley and Young, 2017). Eventually, the degraded products are transported into cytoplasm and recycled for reuse (Klionsky, 2005; Mizushima, 2010). In particular, understanding of the core molecular machinery of autophagy began to be dramatically improved after the identification of the first autophagy-related (Atg) gene, namely, *ATG1*, in *Saccharomyces cerevisiae* (Cheong et al., 2008; Ohsumi, 2014; Yu et al., 2018). To date, more than 40 *ATG* genes have been discovered in yeast cells (Klionsky et al., 2021). Nearly half of yeast *ATG* genes are conserved in mammals, indicating high conservation of the core molecular machine of autophagy across eukaryotes (Galluzzi et al., 2017; Deng et al., 2018). Individual Atg proteins and Atg complexes participate in modulating key events during each stage of autophagy (Yu et al., 2018), whereas dysregulated autophagy with either excessively decreased or increased autophagy activity is closely associated with a broad spectrum of human diseases, such as infectious diseases, neurodegenerative disorders and cancer (Jiang and Mizushima, 2014; Yang and Klionsky, 2020).

Among the 18 core Atg proteins required for autophagosome formation upon nitrogen deprivation in *S*. *cerevisiae* (Xie and Klionsky, 2007; Suzuki et al., 2017; Peng et al., 2021), Atg1 is the only serine/threonine protein kinase (PK) that participates in orchestrating multiple steps in the autophagy pathway (Wang and Kundu, 2017). In the protein sequence, Atg1 contains an N-terminal PK domain, a central intrinsically disordered region (IDR) linker, and an early autophagy targeting and tethering (EAT) domain at the C-terminus (Lin et al., 2018). Thus, Atg1 can either physically interact with other protein partners or covalently phosphorylate protein substrates to regulate autophagy. For example, Atg1 interacts with Atg13, Atg17, Atg29 and Atg31 to form the Atg1 complex, which is activated at the beginning of autophagy (Stjepanovic et al., 2014; Hurley and Young, 2017). The non-covalent interaction of Atg1 with Atg13 or Atg17 is medicated by the EAT domain (Kamada et al., 2000; Kabeya et al., 2005; Cheong et al., 2008). During the autophagic process, Atg1 interacts with Atg8 via the IDR domain, and is translocated with autophagosomes to the vacuole, resulting in degradation of the Atg1 complex (Kraft et al., 2012). On the other hand, the PK activity of Atg1 is also crucial for regulation of autophagy (Mizushima, 2010). The transmembrane protein Atg9 is directly phosphorylated by Atg1, and this phosphorylation event is critical for autophagosome formation in the early step of autophagy (Papinski et al., 2014). Furthermore, Atg1 phosphorylates Atg4 to govern autophagosome maturation (Sanchez-Wandelmer et al., 2017), and phosphorylates Atg29 to sustain autophagy activity (Hu et al., 2019). More recently, it was demonstrated that Atg1 participates in modulating the fusion of autophagosome and vacuole by directly phosphorylating Ykt6, a subunit of the soluble *N*-ethylmaleimide sensitive factor attachment protein receptor (SNARE) complex (Barz et al., 2020). In addition to known Atg proteins and autophagy regulators, Atg1 also interacts with or phosphorylates other proteins in *S*. *cerevisiae*. It is still unclear that how many Atg1-interacting partners and/or phosphorylated substrates are also involved in regulating autophagy.

In this study, we profiled Atg1-dependent molecular landscapes during nitrogen starvation-induced autophagy, by conducting a time-course multi-omic profiling in *S*. *cerevisiae*. Besides differential expression (DE) analysis, we developed a new artificial intelligence (AI) architecture, inference of autophagy regulators from multi-omic data (iAMD), showing area under the curve (AUC) values of 0.874 and 0.810 for predicting potential autophagy regulators from Atg1-interacting partners and substrates, respectively. Based on iAMD predictions, we validated that two proteins, Rgd1 and Whi5, are essential for sustaining bulk autophagy activities in the context of Atg1. Further experiments demonstrated that two phosphorylation sites (p-sites), pS78 and pS149 of Whi5, are phosphorylated by Atg1 and required for maintaining autophagy activity. Moreover, the phosphorylation of Whi5 was validated to regulate the formation of Atg1 puncta during autophagy initiation. Finally, we used iAMD to accurately predict Atg proteins and autophagy regulators from the interactomes of other PKs involved in autophagy, such as target of rapamycin kinase complex I (TORC1), Gcn2, and Yak1, supporting the high transferability of the method. Taken together, we not only revealed two predicted candidates as new autophagy regulators from the Atg1 interactome, but also provided a highly useful resource for further analysis of yeast autophagy.

## 2 Materials and methods

### 2.1 Yeast strains and plasmids

The WT haploid yeast strain was BY4741 (*MATa his3D leu2D met15D ura3D*). The KO mutant strains of the BY4741 background were obtained from a set of yeast deletion clones (Thermo Fisher Scientific, Mat-A Complete Set, 95401.H2). The KO mutants available in this study are individually shown in Supplementary Table S1A. Plasmids expressing Atg1-GFP, GFP-Atg8, Atg17-2×mCherry, Atg1 or Atg1^D211A^ tagged with FLAG, the plasmids for the construction of fusing fluorescent tag GFP with various Atg proteins, including Atg2, Atg5, Atg9, Atg11, Atg13, Atg18 and Atg38, were generated from a previous study (Yi et al., 2017). The plasmid expressing Sec7-2×GFP, Atg1-tdTomato, Atg1 with V5 tag, and the plasmid pCLHN-Nat were obtained from Prof. Zhiping Xie’s laboratory (Shanghai Jiao Tong University) (Zhu et al., 2019; Peng et al., 2021). To generate endogenous protein Rgd1 or Whi5 tagged with GFP, a 2×GFP tag was integrated into the C-terminus of *RGD1* or *WHI5* in WT and *atg1∆* cells using the homologous recombination-based method (Li et al., 2015; Peng et al., 2021). In brief, the plasmid Sec7-2×GFP containing the selection marker URA3 was used as a template, and the DNA fragments flanked by short homologous regions were amplified and produced using PCR with *RGD1-*or *WHI5*-specific primers (Supplementary Table S1B). The PCR products were separately transformed into WT or *atg1∆* yeast cells. Then, the DNA fragments containing the 2×GFP coding sequence were C-terminally integrated at the locus of *RGD1* or *WHI5* through homologous recombination. pEGH plasmids separately expressing Rgd1-HA, Whi5-HA or Whi5-2A-HA were developed in this study, as previously described (Peng et al., 2021). To construct *E. coli* expression vectors of intact Whi5 and Whi5 mutant, the sequences of WT Whi5 or Whi5-2A were separately amplified from yeast expression vector of pEGH-Whi5 or pEGH-Whi5-2A, and then cloned into pCold-TF backbone vector through restriction enzyme digestion and ligation as described in previous studies (Yao et al., 2023a).

### 2.2 Preparation of cell samples

The WT yeasts, *atg1∆* yeasts and *atg1∆* yeasts expressing Atg1-D211A were cultured in YPD medium (Sangon Biotech, A507022). The yeast cells were grown to OD_600_ = 1.0–1.2 overnight and then transferred to SD-N medium (0.17% yeast nitrogen base without amino acids and ammonium sulfate, 2% glucose) for 0, 1 and 4 h. After nitrogen starvation treatment, the yeasts were harvested by centrifugation and rapidly treated with liquid nitrogen for 20 min. Next, the cell samples were stored at −80 °C and prepared for omics identification.

### 2.3 Extraction of total RNA

The yeast cells were lysed in TRIzol (Thermo Fisher Scientific, 15596026), and extraction of total RNA was performed following the manufacturer’s instructions. First, chloroform was added to the cell lysates (Sinopharm Chemical Reagent Co., Ltd., 10006818). After centrifugation at 12,000 × g for 10 min at 4°C, the upper aqueous phase, including the RNA, was separated and transferred. Then, isopropanol (Sinopharm Chemical Reagent Co., Ltd., 40064360) was added to the aqueous phase. The total RNA precipitate was generated after centrifugation, washed twice with 75% ethanol (Sinopharm Chemical Reagent Co., Ltd., 80176961), and resolved in RNase-free water (Invitrogen, 10977023).

### 2.4 RNA-seq library construction and sequencing

The concentration and quality of the total RNA were determined with a NanoDrop 2000 spectrophotometer (Thermo Fisher Scientific, United States), and the integrity of the RNA was measured by utilizing an Agilent 2100 Bioanalyzer system (Agilent Technologies, United States). An equal amount of high-quality total RNA from each sample was utilized for the construction of RNA-seq libraries using a NEBNext Ultra™ II RNA Library Prep Kit for Illumina (NEB, E7770). Next, the products of the RNA-seq libraries were sequenced with a HiSeq 4000 system (Illumina, San Diego, CA).

### 2.5 Total protein extraction

The harvested yeasts were placed on ice and lysed using lysis buffer (8 M urea; Sigma–Aldrich, 554693) containing Protease Inhibitor Cocktail III (Merck Millipore, 539134) and a high-intensity Ultrasonic Cell Disruptor (SCIENTZ). After centrifugation at 12,000 × g for 10 min at 4°C, the cell fragments were removed, and the supernatants of each sample were collected. The protein concentration was measured using a bicinchoninic acid (BCA) protein assay kit (Beyotime, P0012).

### 2.6 HPLC fractionation

The tryptic peptides were fractionated using high-pH reverse-phase HPLC with a Thermo Fisher Scientific BETASIL C18 column (5 μm particles, 4.6 mm, 250 × 10 mm, SN:10428834). In brief, the peptides were first separated with a gradient of 8%–32% acetonitrile (pH 9.0) over 60 min into 60 fractions. Then, the peptides were combined into nine fractions and dried by vacuum centrifugation.

### 2.7 LC–MS/MS analysis

The tryptic peptides were dissolved in solvent A (0.1% formic acid [Sigma–Aldrich, 27001], 2% acetonitrile [Sigma–Aldrich, 900686]) and directly loaded onto a reversed-phase column (250 mm length, 100 μm i.d.). The peptides were separated with a gradient from 6% to 22% solvent B (0.1% formic acid in 90% acetonitrile) over 38 min, 22%–32% over 14 min, and 32%–80% over 4 min followed by a hold at 80% for 4 min. All steps were performed at a constant flowrate of 450 nL/min on an EASY-nLC 1,200 ultra-performance liquid chromatography (UPLC) system (Thermo Fisher Scientific). The separated peptides were analyzed in a Q Exactive™ HF-X (Thermo Fisher Scientific). The electrospray voltage applied was 2.1 kV. The full MS scan resolution was set to 120,000 for a scan range of 350–1,600 m/*z*. Up to the 20 most abundant precursors were then selected for further MS/MS analyses with 30 s dynamic exclusion. HCD fragmentation was performed at a normalized collision energy (NCE) of 28%. The fragments were detected in the Orbitrap at a resolution of 30,000. The fixed first mass was set as 100 m/*z*. The automatic gain control (AGC) target was set at 1E5, with an intensity threshold of 8.3E4 and a maximum injection time of 60 m.

### 2.8 Database search

The resulting MS/MS data were processed using the MaxQuant search engine (v. 1.5.2.8) (Tyanova et al., 2016a). To construct the reference protein database, the proteome sequence set of *S. cerevisiae* was downloaded from UniProt Consortium, 2019 (https://www.uniprot.org/), which included 6,730 unique yeast protein sequences. The detailed parameters are described below. Trypsin/P was specified as the cleavage enzyme, and up to two missed cleavages were allowed. The mass tolerance for precursor ions was set as 20 ppm in the first search and 5 ppm in the main search, and the mass tolerance for fragment ions was set as 0.02 Da. Carbamidomethylation on Cys was specified as the fixed modification. Acetylation on the protein N-terminus, oxidation on Met and deamidation (NQ) were specified as variable modifications. The instrument type was selected as an Orbitrap. For all the other parameters, the default values were set in MaxQuant (Tyanova et al., 2016a).

### 2.9 RNA-seq data analysis

Raw sequence data were first filtered by a well-known next-generation sequencing (NGS) read preprocessing tools Trimmomatic (version 0.39, github.com/usadellab/Trimmomatic) (Bolger et al., 2014). After filtration, reads were aligned to *S. cerevisiae* reference sequence downloaded from NCBI Genome (www.ncbi.nlm.nih.gov/genome, *S. cerevisiae* (assembly R64)) STAR (version 2.7.3a, https://github.com/alexdobin/STAR) (Dobin et al., 2013). Gene expression values were quantitated with RSEM (version 1.3.1, http://deweylab.github.io/RSEM/), an classical quantitative tool for transcriptome expression (Li and Dewey, 2011).

### 2.10 Data normalization and missing value imputation

The omics data were first filtered on the basis of the proportion of valid values in each row, and transcriptomic data were removed if the proportion of valid values was not 100%. For proteomic and phosphoproteomic data, the lowest accepted proportion of valid values was 70%. Then, the omics data were log_2_-transformed and first normalized by row through the *z*-score method. After the above process was performed, the omics data were normalized again by column using the median-centered *z*-score method across the total mRNAs, proteins or p-sites to correct for sample differences. After normalization, the missing values in the omics data were filled according to a normal distribution model across the entire data dataset.

### 2.11 Detection of DEMs, DEPs and DRPs

Here, we took the WT strain data as the background data and compared them with the transcriptomic, proteomic and phosphoproteomic data of *atg1∆* strains or *atg1∆*-Atg1 KD yeasts to detect the differentially regulated mRNAs, proteins and p-sites at 0, 1 and 4 h. The mRNAs with significantly changed FPKM values (FC > 3 or FC < 1/3) for at least one time point were regarded as potential DEMs (*p* < 0.01). For proteomic data and phosphoproteomic data, the *p* values were calculated using the Model-based Analysis of Proteomic data (MAP) method (Li et al., 2019; Qiu et al., 2021). Unlike conventional statistical tests that rely on replicate-based variance estimates, MAP models technical and systematic errors without requiring biological replicates. The method is based on the hypothesis that proteins with similar intensity levels (within a small window) share approximately the same error characteristics. Specifically, proteins and p-sites are first ranked based on their intensities, and a local error function is constructed by analyzing the distribution of fold changes in neighboring proteins. These local functions are then integrated to generate a global error function that serves as the background noise model. Assuming a standard normal distribution of log-transformed fold changes under the null hypothesis, MAP calculates a *z*-score for each protein and p-site, from which a two-tailed p-value is derived. This strategy allows for robust identification of differentially expressed proteins or p-sites, even in datasets with limited or no biological replicates. The proteins and p-sites with significant fold change intensities (FC > 3 or FC < 1/3; *p* < 0.01) for at least one time point were also regarded as potential DEPs and DRPs.

### 2.12 GO enrichment analysis

For the enrichment analysis of the differentially regulated mRNAs, proteins, and p-sites and the 666 known Atg1-interacting proteins, the GO annotation file (released on 16 June 2021) (2019a) was downloaded from The Gene Ontology Consortium, 2019 (http://www.geneontology.org/), and 6,048 yeast genes with at least one annotated GO term were obtained. For each GO term *g*, we defined the following:

*N* = the number of total mRNAs/proteins/phosphoproteins annotated by at least one GO term, *n* = the number of total mRNAs/proteins/phosphoproteins annotated by GO term *g*, *M* = the number of differentially regulated mRNAs/proteins/phosphoproteins or Atg1-interacting proteins annotated by at least one GO term, and *m* = the number of differentially regulated mRNAs/proteins/phosphoproteins or Atg1-interacting proteins annotated by GO term *g*.

Then, the enrichment ratio (E-ratio) of GO term *g* was calculated, and the *p* value was calculated with the hypergeometric distribution, as follows:

$$E‐\mathrm{ratio}=\frac{\frac{m}{M}}{\frac{n}{N}}$$

p value=∑m′=mnMm′N−Mn−m′Nn,E‐ratio > 1.

### 2.13 The iAMD algorithm

There were three steps for implementation of iAMD, including feature encoding, Atg1-interacting model training, and Atg1 substrate model fine-tuning. We described each procedure as below.

1) *Feature encoding*. For each Atg1-interacting protein, its mRNA, protein and phosphorylation levels were taken as the multi-omic features. For WT, *atg1∆*, and *atg1∆*-Atg1 KD strains under nitrogen starvation for 0, 1, and 4 h, we defined the expression vectors of a protein *x* as follows:

$$V_{WT}=Expression\left[{WT}_{0h}{,WT}_{1h}{,WT}_{4h}\right]$$

$$V_{delta}=Expression\left[{atg1∆}_{0h}{,atg1∆}_{1h}{,atg1∆}_{4h}\right]$$

$$V_{KD}=Expression\left[{atg1∆-Atg1\;KD}_{0h}{,atg1∆-Atg1\;KD}_{1h}{,atg1∆-Atg1\;KD}_{4h}\right]$$

From transcriptomic, proteomic and phosphoproteomic data, we obtained the FPKM value, normalized protein intensity, or the sum of normalized p-site intensities if multiple p-sites were quantified in the protein *x*. Then for the protein *x*, the full encoded vectors at mRNA, protein and phosphorylation levels were separately defined as below:

$$V_{mRNA}=FPKM\left[V_{WT}{,V}_{delta}{,V}_{KD}\right]$$

$$V_{Pro.}=Normalized\;intensity\left[V_{WT}{,V}_{delta}{,V}_{KD}\right]$$

$$V_{Phos.}=Sum\;of\;normalized\;intensities\left[V_{WT}{,V}_{delta}{,V}_{KD}\right]$$

To encode sequence data, the frequencies of 20 typical amino acids (alanine [A], arginine [R], …, valine [V]) were counted for the protein *x* in the alphabetical order as below:

$${PseAAC}_{x}={\left(F_{A}{,F}_{R}{,F}_{N},\cdots ,F_{Y},F_{V}\right)}_{20}$$

2) *Atg1-interacting model training*. The four encoded vectors, *V*_*mRNA*_, *V*_*Pro.*_, *V*_*Phos.*_, and *PseAAC*_*x*_ were taken as the informative features for individual model training, using the DNN framework. To reduce overfitting, here we designed a lightweight DNN framework consisting of four layers, including an input layer, two hidden layers, and an output layer. Each layer contained a certain number of computational units named neurons (Supplementary Table S2A). Dropout was implemented after two hidden layers, which randomly dropped some nodes from hidden layers if the accuracy increased. In each layer, all neurons consist of an internal feature representation, receiving input and exporting output. For each neuron in the input layer, a received vector *x* was transformed by the rectified linear unit (ReLU) activation function, which is defined as follows:

$$ReLU\left(x\right)=\left\{\begin{array}{c} x,x\geq 0\\ 0,x<0 \end{array}\right.$$

The first hidden layer was employed for feature extraction and representation, and the second hidden layer was utilized for generating predictions. Nodes in each hidden layer are also activated by the ReLU activation function. The output layer contains two sigmoid neurons adopted to calculate a *score* for the protein *x*, defined as:

$$score\left(x\right)=sigmoid\left(x\right)=\frac{1}{1+e^{-x}}$$

The calculated score, ranging from 0 to 1, represents the probability of the protein *x* to be involved in regulating autophagy or not.

For each Atg1-interacting protein, four scores were calculated by their corresponding DNN models, and used as the secondary features for PLR integration (Shu et al., 2020). Here, we defined the score *S* for the protein *x* as below:

$$S=\left[S_{p}{*W}_{p}\;{+S}_{m}{*W}_{m}+S_{ph}{*W}_{ph}+S_{s}{*W}_{s}\right]$$

Where *S*_*p*_, *S*_*m*_, *S*_*ph*_, and *S*_*s*_ denote the scores from the proteomic, transcriptomic, phosphoproteomic, and sequence models, respectively. *W*_*p*_, *W*_*m*_, *W*_*ph*_, or *W*_*s*_ represent the weight of each score. If the value of *S*_*x*_ (*x* = *p*, *m*, *ph*, or *s*) was not available, we arbitrarily set it as 0.5. The initial *W*_*x*_ was set as 1.

3) *Atg1 substrate model fine-tuning*. In contrast to *de novo* training, the Atg1-interacting model was adopted for fine-tuning an Atg1 substrate model, using the Meta-learning strategy (Finn et al., 2017). Meta-learning is a widely used data augmentation method based on a small amount of training data (Finn et al., 2017). Here, the typical meta-learning algorithm, Model-Agnostic Meta-Learning (MAML), was used. First, the negative data were randomly sampled with a ratio of 1:1 to the positive data. Then, the negative data were mixed with the positive data, and input into the DNN models for training, after feature encoding. The above process was iteratively repeated until the AUC value was not increased any longer.

In both models, predicted proteins with a score ≥0.9 were considered as potential autophagy regulators. For model training, the DNN framework was implemented in the Keras 2.4.3 library (http://github.com/fchollet/keras) with the TensorFlow 2.4.1 backend. During the training process, adjustable parameters including the loss function, optimizer, dropout probability, and mini-batch size were simultaneously optimized to improve the performance (Supplementary Table S2A). The PLR classifier was built on the open Python library of Scikit-learn 0.24.1 (https://scikit-learn.org/stable/index.html) and adjustable parameters were optimized to improve the performance (Supplementary Table S2A). All computational models were trained in a computer with an NVIDIA GeForce GTX 960 GPU, an Intel(R) Core™ i7-6700K @ 4.00 GHz central processing unit (CPU), and 32 GB of RAM.

### 2.14 Performance evaluation and comparison

To evaluate the accuracy of iAMD, the true positive (*TP*), true negative (*TN*), false positive (*FP*) and false negative (*FN*) values were counted for each predictive model. Then, 4 measurements, including the *Sn*, *Sp*, accuracy (*Ac*), and Mathew correlation coefficient (*MCC*), were calculated as follows:

$$Sn=\frac{TP}{TP+FN}$$

$$Sp=\frac{TN}{TN+FP}$$

$$Ac=\frac{TP+TN}{TP+FP+TN+FN}$$

$$MCC=\frac{\left(TP\times TN\right)-\left(FN\times FP\right)}{\sqrt{\left(TP+FN\right)\times \left(TN+FP\right)\times \left(TP+FP\right)\times \left(TN+FN\right)}}$$

For each model, the average *Sn*, *Sp*, *Ac*, and *MCC* values were calculated from the 5-fold cross-validation (Supplementary Table S2B). The ROC curve was illustrated based on the final *Sn* and 1-*Sp* scores, and the AUC value was computed. For each AUC value, the 95% confidence interval was calculated with 1,000 stratified bootstrap replicates (Shu et al., 2020).

To exhibit the superiority of iAMD, which uses multi-omic data and sequence features, over DNN models that are individually trained by single features, we compared the iAMD framework with a proteome-trained DNN model, a transcriptome-trained DNN model, a phosphoproteome-trained DNN model, and a sequence feature-trained DNN model. Also, based on multi-omic data and sequence features, we compared iAMD to four conventional machine learning algorithms, including PLR, SVM, GNB, and RF. The above methods were also implemented in the open source Python library Scikit-learn 0.24.1 (https://scikit-learn.org/stable/index.html). For the Atg1 substrate model, the hybrid-learning model without the meta-learning strategy was also trained. The ROC curves of those models were illustrated, and the AUC values were calculated.

### 2.15 SHAP for model interpretation

Here, the SHAP method was utilized to evaluate the contributions of different features used in iAMD (Lundberg and Lee, 2017; Yuan et al., 2022). Briefly, the SHAP method was used to calculate the contribution scores of the four features for the Atg1-interacting and Atg1 substrate models, respectively, using the open Python library of shap 0.41.0 (https://github.com/slundberg/shap).

### 2.16 RNA isolation and RT–PCR

WT and *atg1∆* cells were inoculated in YPD medium, grown to OD_600_ = 0.8–1.0, and treated with SD-N medium for 0, 1 and 4 h. The cells were collected and lysed to extract the total RNA as previously described (Peng et al., 2021). Total RNA was used as the template to synthesize cDNA using a PrimeScript RT Reagent Kit with gDNA Eraser (Takara, RR047A), and RT–PCR was conducted using AceQ qPCR SYBR Green Master Mix (Vazyme, Q141-02) on a StepOne Real-time PCR system (Thermo Fisher Scientific). The expression level of AFT10 was used as a control, and the relative expression of the targeted genes was calculated by the ∆Ct method as described in previous studies (Peng et al., 2017; Peng et al., 2021). The specific primers for the amplification of each gene are shown in Supplementary Table S1C.

### 2.17 Culture of yeast cells for screening

Plasmids expressing GFP-Atg8 were individually transformed into yeast cells. The WT and KO mutant yeasts were cultured in SD medium (0.17% yeast nitrogen base w/o amino acids and ammonium sulfate, 0.5% ammonium sulfate and 2% glucose) with appropriate dropout (DO) supplements at 30°C overnight. After that, the yeast cells were transferred into SD-N medium for 1 h and then harvested by centrifugation.

### 2.18 GFP-Atg8 immunoblotting assay

The yeasts were collected and lysed using a total protein extraction kit for microbes with thick walls (Minute, YT-015) and protease inhibitor (Roche, 4693159001). The protein concentration was measured with a BCA protein quantification kit (Vazyme, E112). The prepared protein samples were separated by SDS–PAGE and then transferred to polyvinylidene difluoride (PVDF) membranes (Millipore, IPVH00010) under wet conditions. The membranes were incubated with blocking buffer containing 5% nonfat milk (Sangon Biotech, A600669) in 1×TBST (19.8 mM Tris base, 150 mM NaCl, 0.1% Tween-20) solution and probed and analyzed with appropriate antibodies. The antibody for detecting GFP was purchased from Roche (11814460001), the antibody for Pgk1 was purchased from Abcam (ab113687), and the donkey anti-mouse secondary antibody was obtained from LI-COR Biosciences (926-32212). The intensity of each protein band was measured by utilizing an Odyssey_CLx imaging system (LI-COR Biosciences).

### 2.19 GFP-Atg8 fluorescence assay

Yeast cells expressing GFP-Atg8 were grown to OD_600_ = 0.8–1.0 in SD medium supplemented with appropriate DO. Then, the dye FM 4–64 (Invitrogen, T3166) was used to probe the vacuolar membranes of yeasts at a concentration of 25 μg/mL for 30 min. Next, the yeast cells were transferred into rich medium for 30 min at 30°C and then incubated in SD-N medium for 0, 1 and 2 h. To visualize the GFP molecules accumulated in the vacuoles, a confocal microscope (Olympus, FV-3000) was used to observe GFP-Atg8 vacuolar delivery at room temperature.

To quantitatively measure the autophagy activity of yeasts, our recently developed software, DeepPhagy (Zhang et al., 2019), was employed to automatically recognize the GFP signals derived from GFP-Atg8 in the vacuoles. Briefly, three pictures were independently captured for WT, *rgd1*∆ and *whi5*∆ cells expressing GFP-Atg8 at each time point. Next, the obtained images were imported and analyzed to evaluate autophagy activity, which was represented as the ratio of autophagic cells to all recognized cells.

### 2.20 Pho8∆60 assay

DNA fragments containing the antibiotic resistance gene Nat were generated using the plasmid pCLHN-Nat as a PCR template and specific primers for *RGD1* or *WHI5*. The PCR products were transformed into the TN124 strain to construct *rgd1∆* and *whi5∆* mutants through the homologous recombination method (Noda and Klionsky, 2008; Li et al., 2015). The detailed primers are presented in Supplementary Table S1D. In brief, yeast cells were grown to OD600 = 0.8–1.0 in YPD medium at 30°C overnight and treated with SD-N medium for 0 and 4 h. The collected cells were suspended in lysis buffer (20 mM PIPES, pH 6.8, 50 mM KCl, 100 mM potassium acetate, 10 mM MgSO_4_, 10 μM ZnSO_4_, 1 mM PMSF, 0.5% Triton X-100 [Sigma–Aldrich, X100]) by adding glass beads and adequately vortexed for cell disruption. The cell lysates were mixed with reaction buffer (250 mM Tris-HCl, 0.4% Triton X-100, 10 mM MgSO_4_, 10 μM ZnSO_4_) including ρ-nitrophenyl phosphate (Sangon Biotech, A610365), and then stop buffer (1 M glycine-KOH, pH 11.0) was added. The absorbance at 405 nm was determined using a microplate reader (Thermo Fisher Scientific, Multiskan FC), and the protein concentration was detected with a BCA protein quantification kit (Vazyme, E112).

### 2.21 Co-IP assays

Yeast cells expressing Atg1 tagged with FLAG were transformed with plasmids expressing HA-tagged Rgd1 or HA-tagged Whi5. Then, yeasts were cultured to OD_600_ = 0.8–1.0 in SD medium with a final concentration of 2% galactose (Aladdin, G100367) at 30°C overnight and disrupted using a total protein extraction kit for microbes with thick walls (Minute, YT-015) with protease inhibitor (Roche, 4693159001). The cell lysates were incubated with anti-HA agarose beads (Sigma–Aldrich, A2095). Next, the beads were washed using low-salt lysis buffer (50 mM HEPES, pH 7.4, 150 mM NaCl, 1 mM EDTA, 1.5 mM MgCl_2_, 10% glycerol, 1% Triton X-100). The denatured proteins were separated by SDS–PAGE, transferred to PVDF membranes, and finally analyzed with appropriate antibodies. The antibody for detecting HA was purchased from Roche (12013819001), and the antibody for detecting FLAG was purchased from Sigma–Aldrich (A8592). Images of the protein bands were captured by a ChemiDoc XRS + System (Bio–Rad) using Clarity Western ECL Substrate (Bio–Rad, 17-5060).

To validate the potential interaction of Atg1 with Rgd1 or Whi5 during autophagy, the cells expressing V5-tagged Atg1 and HA-tagged Rgd1 or HA-tagged Whi5 were generated, and then grown to OD_600_ = 0.8–1.0 in culture medium supplemented with 2% galactose (Aladdin, G100367) at 30°C overnight. The yeasts were incubated in SD-N medium for 0, 1, 2, 3, and 4 h, respectively. The cell samples were lysed with the protein extraction kit for microbes with thick walls (Minute, YT-015), and cell lysates were incubated with anti-HA agarose beads (Sigma–Aldrich, A2095). The protein immunoprecipitates were separated in SDS-PAGE gel, and finally analyzed with indicated antibodies. The antibody for measuring V5 tag was from Cell signaling (#13202).

### 2.22 Confocal microscope

The cells expressing Atg1-tdTomato and Rgd1 or Whi5 tagged with GFP were cultured and grown to OD600 of 0.6 at 30°C overnight, and then treated with SD-N medium for 0, 1, 2, 3, and 4 h, respectively. The confocal laser scanning microscope (Olympus, FV3000) was utilized to observe the yeast cells through using an UPlanSApo 100×/1,40 Oil DIC. A 488 nm laser and a 594 nm laser were adopted for the observation of protein colocalization. The proportion of colocalization was calculated and analyzed from >300 cells in each experiment. All experiments were independently repeated three times. The two-sided t-test was employed for the calculation of statistical significance (*p* < 0.05).

### 2.23 Fluorescence microscopy

For the construction of the yeast strains BY4741 expressing fluorescent tags, the DNA fragments harboring fluorescent protein GFP were prepared by using restriction enzyme digestion assay, and then separately transformed and integrated into the genome of WT cells and *whi5∆* mutants. The yeast cells stably expressing Atg proteins fused with GFP tag were treated with SD-N medium for 1h, and the images of Atg puncta were captured under the inverted fluorescence microscope (Leica, DMI8). For each experiment, the proportions of cells with fluorescent puncta were measured by calculating 300 cells. All experiments were repeatedly performed with three times. The two-sided t-test was used to evaluate the statistical significance (*p* < 0.05).

### 2.24 Protein purification from *Escherichia coli*

The procedure for protein purification was conducted as previously described (Mao et al., 2021; Yao et al., 2023a). Briefly, *Escherichia coli* BL21 cells (Tsingke, TSV-A09) with His-tag prokaryotic plasmid expressing WT Whi5 or Whi5-2A were inoculated with 400 mL LB medium containing 50 μg/mL of ampicillin (Sangon Biotech, A600064- 0025) and grown to OD_600_ = 0.5–0.6 at 37°C, then cultured with medium with 0.1 mM IPTG (Sangon Biotech, A600168-0025) while shaken at 18°C for 20–24 h. After centrifugation, the bacteria were resuspended in 25 mL lysis buffer (50 mM Tris. HCl, pH 7.5, 500 mM NaCl, 1% Triton X-100 [Sangon Biotech, A600198-0500], 1% PMSF [Sangon Biotech, A610425-0025], 1 mM DTT and 20 mM imidazole). Sonication lysis was followed by centrifugation at 13,000 *g* for 30 min. Three successive washes were performed with 20 mL 1 × PBS or lysis buffer. Protein samples were initially eluted with 1.5 mL elution buffer (100 mM iminazole), and further processed with molecular sieve or anion exchange chromatography for producing high-quality purified proteins.

### 2.25 *In vitro* phosphorylation assay

*In vitro* kinase assays were performed as previously described (Mao et al., 2021; Yao et al., 2023a). In Brief, the yeast cells expressing the Atg1-3×Flag or Atg1^D211A^-3×Flag were grown into log phase, and 50 OD yeasts were collected and lysed. After centrifugation, the supernatants were immunoprecipitated with anti-Flag agarose beads (Sigma, A2220). Purified Atg1 WT- 3×FLAG or Atg1 KD-3×Flag was incubated with purified TF-Whi5 or TF-Whi5-2A from *E. coli* in Atg1 kinase buffer (50 mM HEPES. KOH, pH 7.4, 5 mM NaF, 10 mM MgCl2, 1 mM DTT) with 1.0 μL of 10 mM ATP-γ-S (Sigma, A1388) for 30 min at 30°C, after which 1.5 μL of 50 mM p-nitrobenzyl mesylate/PNBM (Abcam, ab138910) was supplemented. After 1.5 h incubation, the reaction samples were stopped by boiling for 5 min using protein loading buffer. The phosphorylation level of TF-Whi5 or Whi5-2A was detected with using the anti-thiophosphate ester antibody (Abcam, ab92570).

### 2.26 Computational re-construction of the Atg1-centered regulatory network

According to the annotations of GO biological processes, the 75 proteins, including 67 known and newly identified Atg1-interacting proteins, as well as 12 additional autophagy effectors influenced by *RGD1* or *WHI5*, were classified into five groups, including response to cellular stress, transcriptional regulation, protein/membrane transport, autophagosome assembly and formation, and vesicle fusion and degradation. Known PPIs among these proteins were integrated from the nine public databases, including BioGRID (Chatr-Aryamontri et al., 2017), DIP (Xenarios et al., 2002), HINT (Das and Yu, 2012), IID (Kotlyar et al., 2019), IntAct (Kerrien et al., 2012), iRefIndex (Razick et al., 2008), Mentha (Calderone et al., 2013), MINT (Licata et al., 2012) and STRING (Szklarczyk et al., 2021). Potential Atg1 substrates were computationally predicted, using our previously developed GPS algorithm (Xue et al., 2008; Wang et al., 2020). In total, we extracted 682 PPIs for the 75 proteins, and the Atg1-centered regulatory network was constructed and visualized with Cytoscape 3.6.1 software package (Shannon et al., 2003).

### 2.27 Prediction of autophagy regulators from the interactomes of other PKs involved in regulating autophagy

Previously, Oliveira et al. conducted a phosphoproteomic quantification in *S. cerevisiae* after shifts in nitrogen sources and rapamycin incubation, and identified various phosphorylation events in response to increased or decreased TORC1 activity (Oliveira et al., 2015). Also, Dokládal et al. quantified yeast phosphoproteomes with or without *GCN2*, and identified eIF2β as new downstream substrates (Dokladal et al., 2021b). Recently, Dokládal et al. further used quantitative phosphoproteomics to analyze potential substrates of multiple PKs downstream of TORC1, such as Rim15, Yak1, Slt2, and Npr1 (Dokladal et al., 2021a). For each of the 6 PKs, its corresponding interactome was obtained from the nine public PPI databases (Xenarios et al., 2002; Razick et al., 2008; Das and Yu, 2012; Kerrien et al., 2012; Licata et al., 2012; Calderone et al., 2013; Chatr-Aryamontri et al., 2017; Kotlyar et al., 2019; Szklarczyk et al., 2021). In total, we obtained 95, 197, 237, 142, 687 and 149 interacting proteins for TORC1, Gcn2, Rim15, Yak1, Slt2, and Npr1, respectively. From the interactome of each PK, 12, 16, 23, 15, 58, and 17 known Atg proteins and autophagy regulators were separately collected and curated from THANATOS database (Supplementary Table S3) (Deng et al., 2018), as positive data to evaluate the performance of the PK-interacting model. In addition, 4, 4, 4, 2, 9 and 9 known substrates involved in regulating autophagy were separately collected for each PK from literature (Supplementary Table S3), as positive data to evaluate the accuracy of the PK substrate model. For each model, other interacting proteins were taken as negative data. Prior to model training, the same normalization was performed for each phosphoproteomic data set. The AUC value of each model was calculated using the 5-fold cross-validation. The full dataset for additional testing was presented in Supplementary Table S3.

### 2.28 Data and code availability

The RNA-seq datasets have been deposited into the NCBI Sequence Read Archive (SRA, https://www.ncbi.nlm.nih.gov/sra), and the dataset identifier is PRJNA791812. The proteomics and phosphoproteomics datasets, including the annotated MS/MS spectra, have been deposited into Integrated Proteome Resources (iProX, http://www.iprox.org/) (Ma et al., 2019), and the dataset identifier is PXD030628. The main code of iAMD in this study has been uploaded to GitHub at: https://github.com/BioCUCKOO/iAMD.

### 2.29 Statistical analysis

Except for the multi-omic identification, all experiments were independently performed three times in this study. The two-sided t-test was employed for statistical analyses. The mean value and standard error of the mean (SEM) were determined from three independently repeated experiments. The error bars represent the SEM, and a *p* value < 0.05 was regarded to indicate statistical significance.

## 3 Results

### 3.1 The whole procedure of this study

**FIGURE 1:**
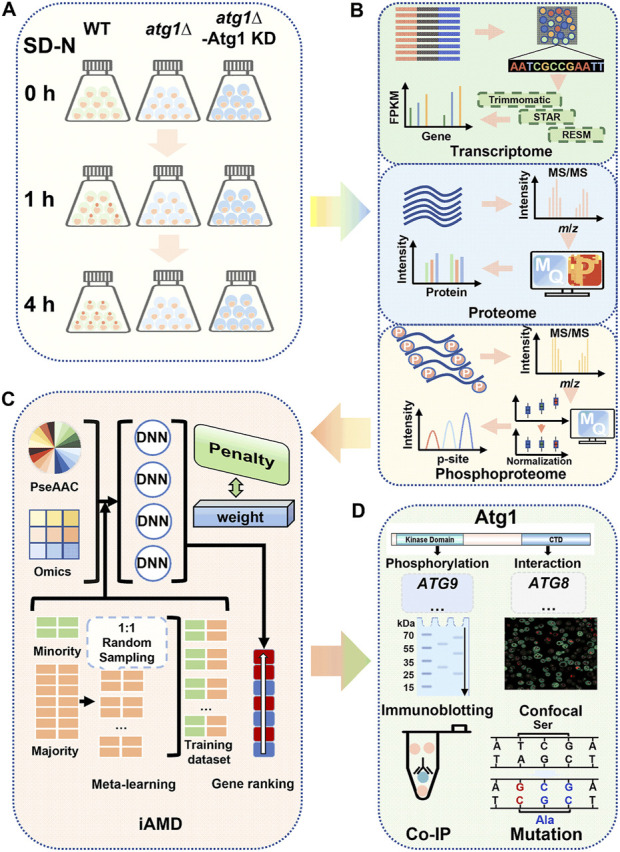
Overall flowchart of this study. **(A)** Preparation of the cell samples. WT, *atg1∆* and *atg1∆* yeast cells expressing the Atg1 kinase-dead (KD) mutant (*atg1∆*-Atg1 KD) were grown and then cultured in SD-N medium for 0, 1, and 4 h. After nitrogen starvation treatment, the cells were harvested for transcriptomics, proteomics and phosphoproteomics. **(B)** Analysis procedure for multi-omic data. For transcriptomics, the RNA-seq data were sequentially analyzed by Trimmomatic, STAR and RSEM for quantification of mRNA expression. MaxQuant was employed for processing of the raw MS/MS data from the proteomics and phosphoproteomics data. **(C)** Method of iAMD. The computational method iAMD was developed to integrate multi-omic datasets and protein sequences, and was used to predict Atg1-interacting partners and substrates that are potentially functional during nitrogen starvation-induced autophagy. **(D)** Experimental validations of the predicted results. Based on the prediction results by using the iAMD framework, additional experiments were performed to validate and confirm the function of new players in autophagy upon nitrogen deprivation.

As a core component of the autophagy machinery, yeast Atg1 physically interacts with a large number of proteins, in which a proportion of these interacting partners can also be phosphorylated by Atg1 through its kinase activity (Mizushima, 2010; Hurley and Young, 2017; Wang and Kundu, 2017). Here, we conducted a multi-omic profiling to analyze the molecular landscapes shaped by Atg1, as well as the prediction and validation of potentially new autophagy regulators from the Atg1 interactome (Figure 1). First, wild-type (WT) cells, *ATG1* knockout (*atg1∆*) cells, and *atg1∆* cells with rescue of the kinase-dead mutant Atg1^D211A^ (*atg1∆*-Atg1 KD) (Yi et al., 2017) in the BY4741 background were grown and cultured in the synthetic minimal medium lacking nitrogen (SD-N) for 0, 1, and 4 h, respectively (Figure 1A). At each time point, the yeasts were collected and prepared for transcriptomic, proteomic and phosphoproteomic quantifications (Figure 1A).

Next, the multi-omic datasets were appropriately processed and analyzed with a series of computational tools (Figure 1B). In this study, WT data were regarded as the control, while DE molecules were determined through pairwise comparisons of *atg1∆* or *atg1∆*-Atg1 KD yeasts to WT yeasts. To identify potential differentially expressed mRNAs (DEMs) from RNA sequencing (RNA-seq) transcriptomic data, a widely applied workflow including Trimmomatic (Bolger et al., 2014), STAR (Dobin et al., 2013), and RSEM (Li and Dewey, 2011) was adopted to align the short reads to the reference genome of *S*. *cerevisiae*, determine the relative expression levels of gene transcripts by calculating the fragments per kilobase of exon per million fragments mapped (FPKM) values, and pairwisely estimate the statistical significance between two yeast strains for each time point (Figure 1B). To determine differentially expressed proteins (DEPs) and differentially regulated p-sites (DRPs), a label-free quantitation strategy combined with the liquid chromatography-tandem mass spectrometry (LC–MS/MS) was employed for proteomic and phosphoproteomic quantification (Figure 1B; Supplementary Figures S1A–C). To process the raw MS/MS spectra, a frequently used computational platform MaxQuant (Tyanova et al., 2016a), was adopted for database search of the reference protein sequences in *S*. *cerevisiae*, and interpretation of the mass spectrometric signals into the intensity values of proteins or p-sites (Figure 1B). To diminish the batch effect during proteomic and phosphoproteomic quantification, another tool Perseus was adopted for data normalization using the *z*-score method (Tyanova et al., 2016b; Shahriyari, 2019). After normalization and imputation of missing values, potential DEPs and DRPs affected by Atg1 during autophagy were finally determined.

Prior to multi-omic data integration, we carefully curated 666 nonredundant Atg1-interacting proteins from nine widely used public databases that contained experimentally identified or computationally predicted protein–protein interactions (PPIs), including eight known Atg1 substrates, such as Atg4, Atg9, Atg29 and Ykt6 (Yeh et al., 2010; Papinski et al., 2014; Kamber et al., 2015; Sanchez-Wandelmer et al., 2017; Hu et al., 2019; Barz et al., 2020; Kira et al., 2021). From the Atg1 interactome, we developed iAMD for predicting new autophagy regulators in the context of Atg1, by integrating the multi-omic features and sequence properties (Figure 1C). To evaluate the accuracy of iAMD, we compiled a benchmark data set of 65 known Atg proteins and autophagy regulators that interact with the yeast Atg1, from a previously developed database termed The Autophagy, Necrosis, ApopTosis OrchestratorS (THANATOS, http://thanatos.biocuckoo.org) (Deng et al., 2018), together with the eight known Atg1 substrates (Yeh et al., 2010; Papinski et al., 2014; Kamber et al., 2015; Sanchez-Wandelmer et al., 2017; Hu et al., 2019; Barz et al., 2020; Kira et al., 2021).

Based on the iAMD predictions, known Atg proteins and autophagy regulators were singled out, and the remaining candidates having corresponding knockout (KO) mutant yeast strains were screened using the green fluorescent protein (GFP)-Atg8 immunoblotting assay (Peng et al., 2021), an extensively applied method for monitoring autophagy activity in yeast cells (Figure 1D). Then, the real-time polymerase chain reaction (RT–PCR) and immunoblotting assays were performed to probe the mRNA and protein changes of screened candidates with or without *ATG1* during autophagy. Furthermore, DeepPhagy, a deep learning-based computational tool for quantitatively monitoring yeast autophagy activity (Zhang et al., 2019), was adopted to evaluate the functional impacts of candidate KO mutants on autophagy activity (Figure 1D). Moreover, the co-immunoprecipitation (Co-IP) assay was employed to validate potentially physical interactions between Atg1 and new autophagy regulators (Figure 1D) (Peng et al., 2021). Using the site-directed mutagenesis method, further validations were conducted to probe functional effects of p-sites potentially modified by Atg1 (Yi et al., 2017). Besides the modeling of an Atg1-centered network to put our findings in the context of the current knowledge, the extendability of iAMD was tested, by accurate predictions of potential substrates of other PKs involved in autophagy.

### 3.2 Characterization of Atg1-dependent molecular landscapes via multi-omic profiling

**FIGURE 2:**
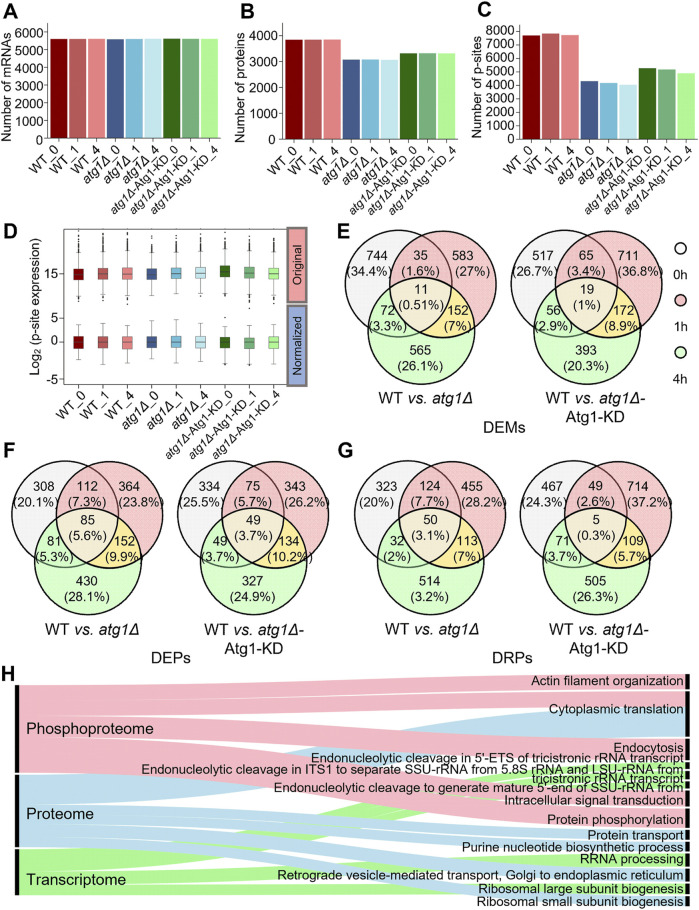
Profiling of the transcriptomics, proteomics and phosphoproteomics data related to autophagy. **(A)** Numbers of mRNAs detected with at least one read in WT, *atg1∆* and *atg1∆*-Atg1 KD samples. **(B)** Numbers of proteins detected with at least one peptide in WT, *atg1∆* and *atg1∆*-Atg1 KD samples. **(C)** Numbers of p-sites detected with at least one peptide in WT, *atg1∆* and *atg1∆*-Atg1 KD samples. **(D)** Distribution of the normalized and unnormalized intensity values for the identified p-sites. **(E)** Overlap of significantly regulated mRNAs between WT and *atg1∆* strains and between WT and *atg1∆*-Atg1 KD strains at three time points. **(F)** Overlap of significantly regulated proteins between WT and *atg1∆* strains and between WT and *atg1∆*-Atg1 KD strains at three time points. **(G)** Overlap of significantly regulated p-sites between WT and *atg1∆* strains and between WT and *atg1∆*-Atg1 KD strains at three time points. **(H)** GO-based enrichment analysis of biological processes at the mRNA, protein and phosphoprotein levels.

From transcriptomic profiling of the nine samples, approximately 2.08 × 10^8^ clean reads were obtained and then mapped to nonredundant yeast mRNAs (Supplementary Figure S1D). Similar mRNA expression levels were presented in the nine samples, and the average FPKM value was 227.82 (Supplementary Figure S1G; Supplementary Table S4). In the results, 5,779 yeast mRNAs were detected in at least one sample, and the average number of yeast mRNAs per sample was 5,605 (Figure 2A). In particular, 5,617 (97.2%) genes were mutually detected in the WT, *atg1∆* and *atg1∆*-Atg1 KD strains, indicating the high reliability of transcriptomic data. As expected, the average FPKM values of *ATG1* in the WT and *atg1∆* strains were 81.00 and 0.08, respectively, demonstrating the nearly complete deletion of *ATG1* in *atg1∆* cells. The FPKM value of *ATG1* was 241.67 in the *atg1∆*-Atg1 KD strain, indicating that the *ATG1* mutant gene was ectopically expressed in the yeasts (Supplementary Table S4). From transcriptomic data, 862, 781, and 800 regulated DEMs were identified at 0, 1 and 4 h between the *atg1∆* and WT strains, using a threshold of fold change (FC) > 3 or <1/3 (*p* < 0.01) (Figure 2E; Supplementary Tables S5A, B). Moreover, we also detected 657, 967, and 640 regulated DEMs at the three time points in *atg1∆*-Atg1 KD yeasts compared to WT cells (FC > 3 or <1/3*, p* < 0.01) (Figure 2E; Supplementary Tables S5A, B). In total, 244 DEMs were found to be potentially regulated by *ATG1* at both the 1 and 4 h during yeast autophagy (Supplementary Figure S1I).

For analysis of proteomic data, 34,649 nonredundant peptides were detected in the nine samples, and mapped to their corresponding protein sequences. Then, 3,993 unique proteins were detected with at least one identified peptide, and the average number of detectable proteins per sample was 3,415 (Figure 2B; Supplementary Table S6). In the results, 3,562 (89.2%) identified proteins were detected with ≥2 peptides (Supplementary Figure S1E). In contrast to transcriptomic data, the overlap of proteins among WT, *atg1∆* and *atg1∆*-Atg1 KD yeasts was considerably reduced to 75.1%. Considering the high dynamics of protein changes and the relative lower coverage of proteomic profiling, such a high overlap among the three yeast cell types still indicated the promising reliability of our proteomic data. After data normalization (Supplementary Figure S1H), we identified 586, 713, and 748 DEPs at 0, 1, and 4 h between the *atg1∆* and WT strains, using the threshold of FC > 3 or <1/3 (*p* < 0.01). Similarly, 507, 601, and 559 DEPs were identified in *atg1∆*-Atg1 KD yeast at the 3 time points compared to WT yeast (FC > 3 or <1/3, *p* < 0.01) (Figure 2F; Supplementary Tables S5C, D). Finally, 245 DEPs were identified to be potentially regulated by Atg1 at both the 1 and 4 h during the autophagic process (Supplementary Figure S1J).

For phosphoproteomic profiling, 13,140 nonredundant p-sites in 2,350 phosphoproteins were obtained, and the average number of identified p-sites in each yeast sample was 5,687 (Figure 2C; Supplementary Table S7). Here, we detected 1,852 (78.8%) phosphoproteins with ≥2 peptides (Supplementary Figure S1F). The overlap of phosphoproteins among the WT, *atg1∆* and *atg1∆*-Atg1 KD yeasts was decreased to 68.2%. Because protein phosphorylation is a dynamically reversible covalent modification, phosphopeptides from each sample were enriched *in vitro* before LC–MS/MS analyses. The remarkably high overlap among the three strains convincingly showed the high data quality of phosphoproteomics. After using the *z*-score method to normalize phosphoproteomic data (Figure 2D), we found 529, 742, and 709 DRPs at 0, 1, and 4 h between the WT and *atg1∆* yeast strains, whereas 592, 877, and 690 DRPs were identified at the three time points in *atg1∆*-Atg1 KD cells compared to WT cells (FC > 3 or <1/3; *p* < 0.01) (Figure 2G; Supplementary Tables S5E, F). Similarly, we observed that 272 DRPs in 217 phosphoproteins might be influenced by Atg1 at both the 1 and 4 h in autophagy (Supplementary Figure S1K).

Next, Gene Ontology (GO)-based enrichment analysis (2019a) was conducted to illustrate the main biological processes that are potentially regulated by *ATG1* according to transcriptomic, proteomic and phosphoproteomic data (Figure 2H). Strikingly, we observed that a number of RNA processing-related biological processes were exclusively enriched at the mRNA level, while the cytoplasmic translation (GO:0002181) was overrepresented at the protein and phosphorylation levels. In particular, the enrichment of protein phosphorylation (GO:0006468) highlights the importance of Atg1 kinase activity during nitrogen starvation-induced autophagy. Thus, our results indicated diverse functions of Atg1 in altering molecular landscapes at the transcriptional, translational and phosphorylation levels.

### 3.3 Dynamic changes of the Atg1 interactome during autophagy

**FIGURE 3:**
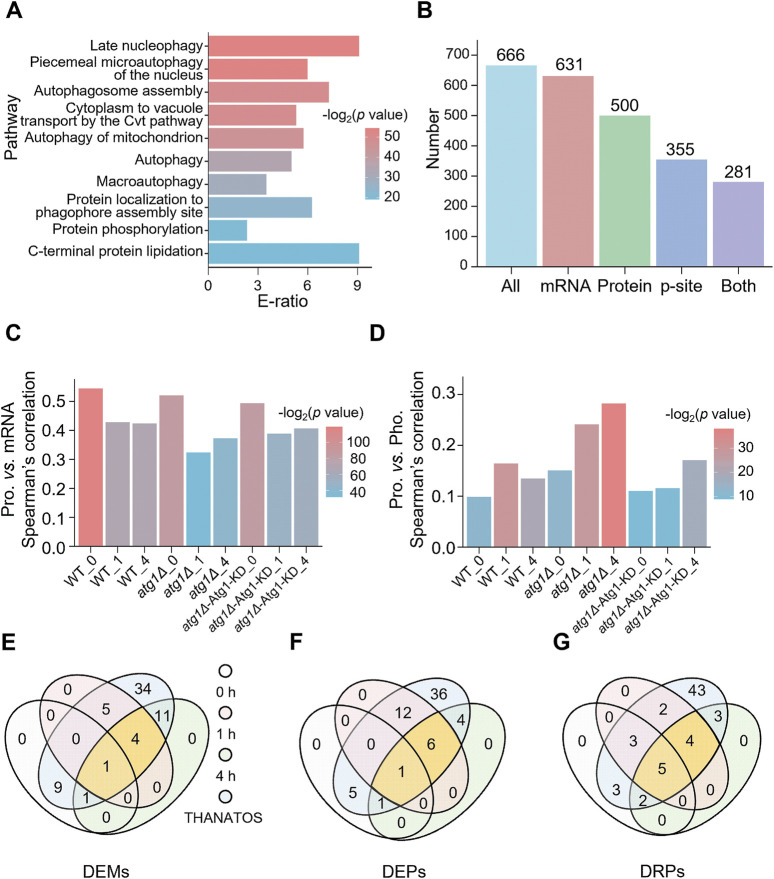
Analysis of Atg1-interacting molecules in yeast autophagy. **(A)** GO-based enrichment analysis of 666 Atg1-interacting proteins based on biological process GO terms. **(B)** Numbers of Atg1-interacting proteins identified from the transcriptomic, proteomic and/or phosphoproteomic analyses. **(C)** Spearman’s correlation between mRNA and protein abundance for the Atg1-interacting proteins. **(D)** Spearman’s correlation between protein and p-site abundance for the Atg1-interacting proteins. **(E)** Overlap of the DEMs in the Atg1 interactome and the 65 known Atg proteins and autophagy regulators in THANATOS (Deng et al., 2018). **(F)** Overlap of the DEPs in the Atg1 interactome and the 65 known Atg proteins and autophagy regulators in THANATOS (Deng et al., 2018). **(G)** Overlap of the DRPs in the Atg1 interactome and the 65 known Atg proteins and autophagy regulators in THANATOS (Deng et al., 2018).

From nine public PPI databases, including BioGRID (Chatr-Aryamontri et al., 2017), DIP (Xenarios et al., 2002), HINT (Das and Yu, 2012), IID (Kotlyar et al., 2019), IntAct (Kerrien et al., 2012), iRefIndex (Razick et al., 2008), Mentha (Calderone et al., 2013), MINT (Licata et al., 2012) and STRING (Szklarczyk et al., 2021), we obtained 666 experimentally identified or computationally predicted Atg1-interacting proteins, including eight known substrates phosphorylated by Atg1 (Supplementary Figure S2A). To investigate the correlation between the Atg1 interactome and autophagy, GO-based enrichment analysis was conducted for the 666 Atg1-interacting proteins. Interestingly, we observed that the majority of overrepresented biological processes have been previously reported to be closely related to autophagy (Figure 3A; Supplementary Table S8A). For example, the most enriched process was late nucleophagy (GO:0044805), a typical selective autophagy pathway (Mijaljica and Klionsky, 2021). Interestingly, autophagosome assembly (GO:0000045), autophagy (GO:0006914) and macroautophagy (GO:0016236) were enriched, supporting the essential role of the Atg1 interactome in orchestrating the autophagic process (Supplementary Table S8).

Next, the 666 Atg1-interacting proteins were mapped to transcriptomic, proteomic and phosphoproteomic data. From the results, 631 (94.7%), 500 (75.1%), and 355 (53.3%) Atg1-interacting partners were quantitatively detected at the mRNA, protein and phosphorylation levels, respectively, whereas 281 (42.2%) genes were simultaneously identified at all the 3 omics levels (Figure 3B). For all the nine samples, the mRNA, protein and phosphorylation levels of the Atg1-interacting partners are visualized in a heatmap (Supplementary Figures S2B–D). In each of the nine samples, the correlation between steady-state mRNA and protein abundance of Atg1-interacting partners was calculated by Spearman’s correlation coefficient (SCC), which ranged from 0.323 to 0.545 (Figure 3C). Obviously, the mRNA-protein abundance correlation was considerably higher in WT yeasts at 0, 1 and 4 h (SCC ≥0.424, *p* ≤ 3.02 × 10^−21^). The mRNA-protein abundance correlation was markedly reduced in *atg1∆* yeasts under nitrogen starvation for 1 h and 4 h, with SCC values of 0.324 (*p* = 3.08 × 10^−10^) and 0.373 (*p* = 2.22 × 10^−13^), respectively (Figure 3C). Due to rescued expression of the Atg1 KD mutant in *atg1∆* cells, the mRNA-protein correlations were increased in *atg1∆*-Atg1 KD yeasts under nitrogen starvation for 1 h and 4 h, with SCC values of 0.389 (*p* = 6.46 × 10^−16^) and 0.407 (*p* = 2.04 × 10^−17^), respectively (Figure 3C). The results suggested that the Atg1-mediated interaction, but not its kinase activity, is mainly responsible for the accordant changes in mRNA and protein expression levels of its interacting proteins during the autophagic process.

Similarly, the correlation between steady-state protein abundance and p-site intensity of Atg1-interacting partners was also calculated for each sample. We observed that the steady-state protein-p-site correlation of Atg1-interacting proteins was significantly increased in *atg1∆* strains treated by nitrogen deprivation for 0, 1, and 4 h, by pairwise comparison with those in WT and *atg1∆*-Atg1 KD yeasts (Figure 3D). Again, this result indicated that the accordant changes in protein and phosphorylation levels of Atg1-interacting proteins were mainly determined by the Atg1-mediated interaction, but not its kinase activity. Taken together, our results proposed that the Atg1-mediated interaction might be a prerequisite in regulating the protein stability of its interacting partners during the autophagic process.

By mapping the 666 Atg1-interacting partners to THANATOS (Deng et al., 2018), we obtained a benchmark dataset of 65 known Atg proteins and autophagy regulators that interact with yeast Atg1. Then, we explored the molecular dynamics of the 65 known Atg proteins and autophagy regulators during autophagy, and observed that more than half of them were not significantly changed at any of the three omic levels (Figures 3E–G). From the results, 4, 6 and 4 known Atg proteins and autophagy regulators with significant changes at both 1 and 4 h were detected at all the three omic levels, respectively (Figures 3E–G).

### 3.4 Development of iAMD for predicting new autophagy regulators

From the above analysis, our results indicated that most of the known Atg1-interacting Atg proteins and autophagy regulators only underwent moderate changes at the mRNA, protein or phosphorylation level during the autophagic process. Thus, the traditional DE analysis will be difficult for identification of new autophagy regulators in the context of Atg1 directly from the multi-omic data. Here, we hypothesized that in the Atg1 interactome, if other interacting partners exhibited similar molecular characteristics to known Atg proteins and/or autophagy regulators, they might have similar functions and also participate in regulating autophagy. From the literature, we also collected eight known Atg1 phosphorylated substrates, and directly training a computational model with such a small data will be highly over-fitting and error-prone.

**FIGURE 4:**
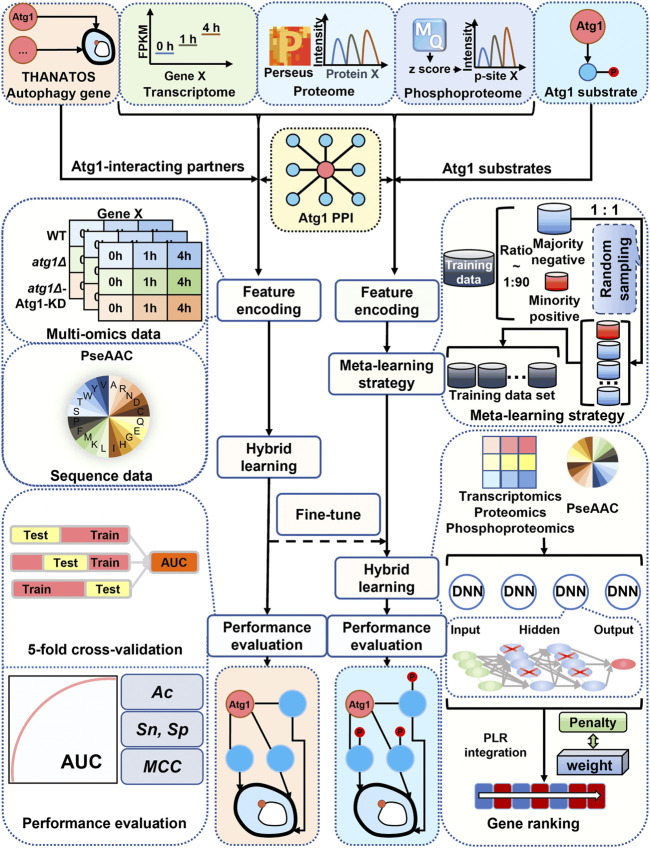
Schematic framework of iAMD. The 666 nonredundant PPIs in *Saccharomyces cerevisiae* were carefully curated from nine public databases, including BioGRID (Chatr-Aryamontri et al., 2017), DIP (Xenarios et al., 2002), HINT (Das and Yu, 2012), IID (Kotlyar et al., 2019), IntAct (Kerrien et al., 2012), iRefIndex (Razick et al., 2008), Mentha (Calderone et al., 2013), MINT (Licata et al., 2012) and STRING (Szklarczyk et al., 2021). For prediction of Atg1-interacting partners potentially involved in autophagy, 65 known Atg proteins and autophagy regulators were used as positive data, and the remaining 601 proteins were as negative data. Then, multi-omic and sequence features were separately trained by the DNN framework, and the output values were integrated by PLR to generate the final predictive score. For prediction of Atg1 substrates potentially involved in regulating autophagy, eight experimentally validated Atg1 substrates were taken as positive data, and the remaining 658 Atg1-interacting proteins were taken as negative data. The Atg1-interacting model was used for fine-tuning an Atg1 substrate model, based on the meta-learning strategy. Finally, after the filtration, the predicted candidates were reserved for further experimental validations.

To accurately and robustly predict both Atg1-interacting partners and substrates that also participate in regulating autophagy, we developed a hybrid-meta learning architecture termed iAMD (Figure 4). First, we incorporated a deep learning framework, deep neural network (DNN), with a traditional machine learning algorithm, penalized logistic regression (PLR), for predicting potential Atg1-interacting partners that are also involved in regulating autophagy. From the 666 curated Atg1-interacting partners, the 65 known Atg proteins and autophagy regulators were taken as positive data, and the remaining 601 proteins were regarded as the negative data (Supplementary Table S8B). For each Atg1-interacting partner, we considered the mRNA, protein and phosphorylation levels, as well as the protein sequence feature of pseudo-amino acid composition (PseAAC) (Ning et al., 2020; Ning et al., 2021), as four types of informative features. In iAMD, we used the DNN to train an initial model for each feature, and four generated scores were adopted as secondary features to be trained by PLR, to obtain an optimized prediction score.

Then, a small-sample learning strategy, meta-learning (Finn et al., 2017), was further adopted to predict potential Atg1 substrates that are also involved in regulating autophagy. Due to the data limitation, we only collected eight known Atg1 substrates as positive data, including Atg1 (Yeh et al., 2010), Atg2 (Papinski et al., 2014), Atg4 (Sanchez-Wandelmer et al., 2017), Atg6 (Kamber et al., 2015), Atg9 (Papinski et al., 2014), Atg13 (Kira et al., 2021), Atg29 (Hu et al., 2019), and Ykt6 (Barz et al., 2020), from the published literature. The remaining 658 Atg1-interacting partners were taken as negative data for this learning task (Supplementary Table S8B). In contrast to *ab initio* train a model, we employed meta-learning to fine-tune the model of predicting Atg1-interacting partners, using the dataset of Atg1 substrates.

### 3.5 Performance evaluation and comparison of iAMD to other methods

**FIGURE 5:**
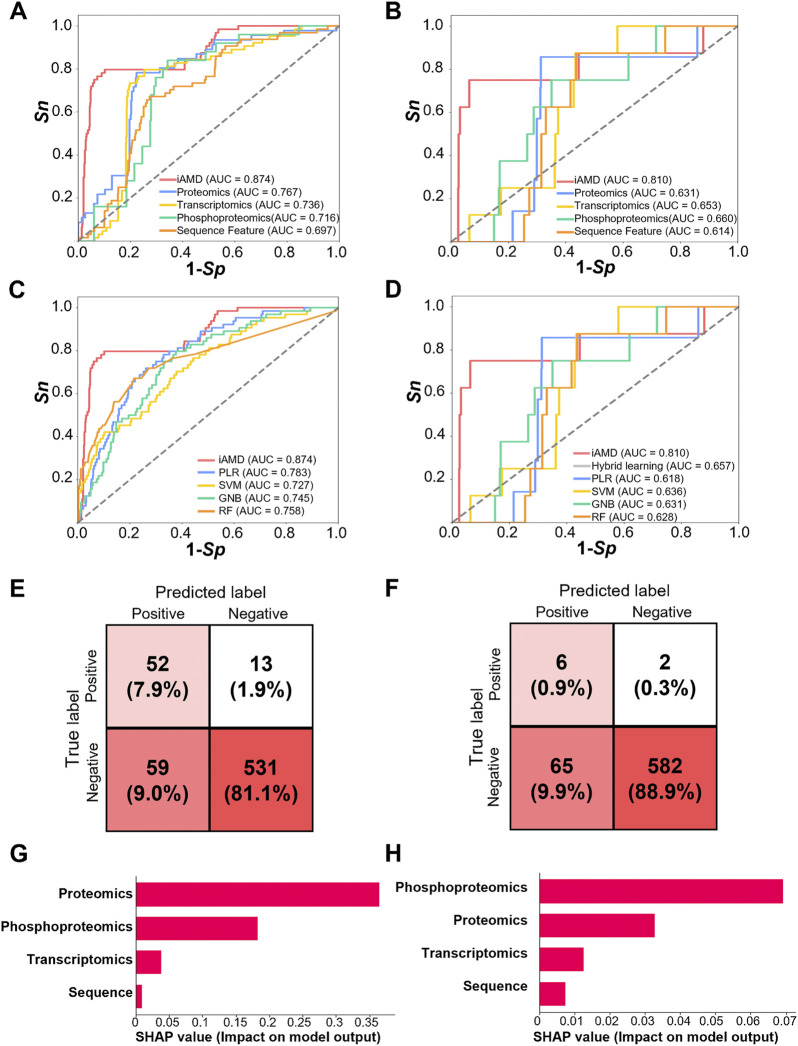
Performance and comparison of iAMD and other methods. **(A)** The AUC values were calculated for Atg1-interacting partner prediction methods including iAMD and single feature-trained DNN models. **(B)** The AUC values were calculated for Atg1 substrate prediction methods including iAMD and single feature-trained DNN models. **(C)** AUC values of the Atg1-interacting partner prediction methods including iAMD, PLR, SVM, GNB, and RF. **(D)** AUC values of the Atg1 substrate prediction methods including iAMD, a hybrid learning method without meta-learning, PLR, SVM, GNB, and RF. **(E)** Confusion matrix of the prediction model of Atg1-interacting partners in the iAMD framework. **(F)** Confusion matrix of the prediction model of Atg1 substrates in the iAMD framework. **(G, H)** The SHAP values of the features contributed for the Atg1-interacting or Atg1 substrate model.

To evaluate the accuracy of iAMD, sensitivity (*Sn*) and specificity (*Sp*) were calculated from the 5-fold cross-validation. The receiver operating characteristic (ROC) curves were illustrated, and AUC values were calculated as 0.874 (95% confidence interval [CI] = 0.820–0.922) for the Atg1-interacting model (Figure 5A) and 0.810 (95% CI = 0.787–0.831) for the Atg1 substrate model (Figure 5B). To exhibit the superiority of iAMD, we compared its performance with each of the individual DNN models using a single feature. For prediction of Atg1-interacting partners involved in regulating autophagy, our results indicated that the AUC values of the proteome-, transcriptome-, phosphoproteome- and PseAAC-based DNN models were 0.767, 0.736, 0.716 and 0.697, respectively (Figure 5A). For prediction of Atg1 substrates involved in regulating autophagy, the AUC values of the proteome-, transcriptome-, phosphoproteome- and PseAAC-trained DNN models were 0.631, 0.653, 0.660 and 0.614, respectively (Figure 5B). By comparison, iAMD showed >14.0% and >27.7% higher AUC values for the Atg1-interacting and substrate models, respectively. Thus, our results supported the advantage of the hybrid-meta learning architecture that integrated multiple features.

To further exhibit the superiority of iAMD, a number of traditional machine learning algorithms, including the PLR, support vector machine (SVM), Gaussian naïve Bayes (GNB), and random forest (RF) algorithms, were utilized for model training and comparison. In particular, a hybrid-learning model without meta-learning was also implemented for the Atg1 substrate model. Through the 5-fold cross-validation, the AUC values of PLR, SVM, GNB, and RF were calculated as 0.783, 0.727, 0.745, and 0.758, respectively, for prediction of Atg1-interacting partners involved in regulating autophagy (Figure 5C). By comparison, iAMD achieved an >11.6% higher AUC value (0.874 vs. 0.783). For prediction of Atg1 substrates involved in regulating autophagy, the AUC values of PLR, SVM, GNB, RF and hybrid learning without meta-learning were 0.618, 0.636, 0.631, 0.628 and 0.657, respectively (Figure 5D). The AUC value of hybrid learning was only 3.3% higher than that of SVM (0.657 vs. 0.636), whereas the AUC value of iAMD further using meta-learning was increased to 0.810. The results indicated that the hybrid-meta learning framework of iAMD exhibited the superior performance compared with other machine learning algorithms. Under a threshold of *Sp* = 90%, confusion matrices were illustrated for the Atg1-interacting and substrate models, respectively, supporting the accuracy and reliability of iAMD (Figures 5E, F).

To further evaluate differential contributions of the four features in iAMD, a widely used method, namely, SHapley Additive exPlanation (SHAP) (Lundberg and Lee, 2017; Yuan et al., 2022), was employed for model interpretations. From the results, it was observed that all the four features contributed for the two predictive models (Figures 5G, H). For the Atg1-interacting model, the SHAP value of the proteomic feature is the highest, indicating the importance of protein expression for predicting Atg1-interacting proteins involved in autophagy (Figure 5G). For the Atg1 substrate model, the phosphoproteomic feature showed the most important contribution for the predictive model, and the result was consistent with the importance of Atg1 kinase activity in modifying their substrates (Figure 5H). Taken together, our analyses not only showed the superior and robust performance of iAMD, but also revealed the most informative feature for each computational model.

### 3.6 Prediction of Atg1-interacting proteins and substrates potentially functional in autophagy

**FIGURE 6:**
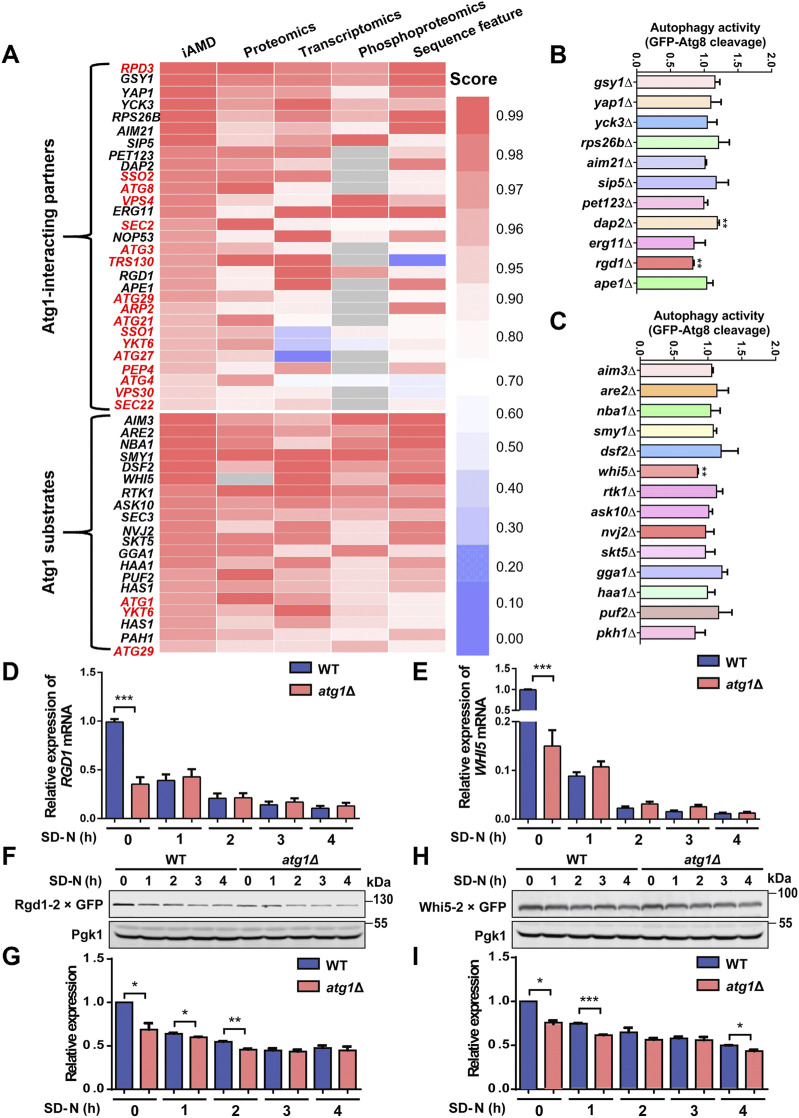
Computational predictions for the players that are potentially functional during autophagy. **(A)** The 29 Atg1-interacting partner candidates and 20 Atg1 substrate candidates predicted to be potentially functional in autophagy upon nitrogen starvation stimulation, including 17 known Atg proteins and autophagy regulators and three known Atg1 substrate marked in red. The scores indicate their probabilities of being real Atg1-interacting partners or Atg1 substrates. **(B, C)** Functional screening for 25 genes using their corresponding KO mutant strains. WT and KO mutant cells expressing GFP-Atg8 were treated with SD-N medium for 1 h, and the cleavage of GFP-Atg8 was detected by immunoblotting. The ratio between free GFP and the total amounts of free GFP and GFP-Atg8 in each KO mutant strain was calculated and compared with that of the WT strain. **(D, E)** The mRNA levels of *RGD1* and *WHI5* in WT and *atg1∆* cells treated with SD-N medium for 0, 1, 2, 3, and 4 h. **(F, G)** WT and *atg1∆* cells expressing Rgd1-2×GFP were cultured under nitrogen starvation conditions for 0, 1, 2, 3, and 4 h. Denatured proteins were detected by immunoblotting using anti-GFP antibodies. The relative expression of Rgd1 was determined according to the ratios between Rgd1-2×GFP and Pgk1. **(H, I)** The protein levels of Whi5-2×GFP were measured and determined in WT and *atg1∆* yeasts after nutrient deprivation for 0, 1, 2, 3, and 4 h. *p < 0.05; **p < 0.01; ***p < 0.001.

Using iAMD, 29 Atg1-interacting partners and 20 substrates were finally prioritized with a stringent cutoff value (scores ≥0.9) (Figure 6A; Supplementary Tables S2B, C). After mapping these candidates to the known Atg proteins and autophagy regulators in the THANATOS database (Deng et al., 2018), we reserved 12 Atg1-interacting partners and 17 Atg1 substrates not reported to be involved in regulating autophagy. For the remaining 29 candidates, we carefully checked the library of yeast deletion clones and found that 11 and 14 genes among the interacting partners and substrates had corresponding KO strains, respectively.

For the 25 candidate genes, WT and KO yeast cells were separately transformed with the GFP-Atg8 expression plasmid and then treated under nitrogen starvation for 1 h. The ratios between free GFP and the total amounts of free GFP and GFP-Atg8 were calculated to evaluate autophagy activity in each yeast strain. Through functional screening, our results showed that autophagy activity was significantly altered in three KO mutants, *dap2∆*, *rgd1∆* and *whi5∆*, compared to the WT strains (Figures 6B, C). Because some important proteins that are related to autophagy might be neglected in THANATOS, we re-curated the published literature to confirm whether the three genes were previously reported to play regulatory roles in autophagy. As a known autophagy regulator, Dap2 is a vacuolar hydrolase, and its enzymatic activity is involved in the degradation of proteins destined for vacuoles through various trafficking pathways, such as autophagy and the cytoplasm-to-vacuole targeting (Cvt) pathway (Parzych and Klionsky, 2019). However, Rgd1 is annotated as a Rho GTPase-activating protein that upregulates the GTPase activities of Rho3 and Rho4, and is critical for the maintenance of cell polarity in eukaryotes (Lefebvre et al., 2009; Lefebvre et al., 2012). Also, Whi5 is a transcriptional repressor that binds to and suppresses SCB binding factor (SBF) transcriptional complexes during early G1 of the cell cycle and plays an important role in modulating cellular growth and division (Takahata et al., 2009; Schmoller et al., 2015; Qu et al., 2019; Barber et al., 2020). Both Rgd1 and Whi5 were not reported to be involved in autophagy, and whether they could act as potential autophagy regulators need to be further explored.

To test whether Atg1 affects the mRNA and protein expression levels of Rgd1 and Whi5 during the autophagic process induced by nitrogen starvation, we first used the RT–PCR assay to detect the mRNA expression of *RGD1* and *WHI5* in WT and *atg1*∆ cells treated with SD-N medium for 0, 1, 2, 3 and 4 h, respectively. The mRNA expression levels of *RGD1* and *WHI5* were markedly decreased only at 0 h in *atg1*∆ cells compared to WT cells (Figures 6D, E), indicating both proteins were not affected by *ATG1* at the transcriptional level during autophagy. Next, to investigate the effect of Atg1 on the endogenous protein expression of Rgd1 and Whi5 in autophagy, we employed a homologous recombination-based method (Klionsky, 2005; Qu et al., 2019) to individually integrate a C-terminal 2×GFP tag at the *RGD1* or *WHI5* locus in WT and *atg1*∆ cells, respectively. Yeast cells expressing the GFP tag were treated under nitrogen starvation for 0, 1, 2, 3 and 4 h, respectively. The immunoblotting results showed that the expression levels of Rgd1-2×GFP were significantly lower in *atg1*∆ cells at 0, 1, and 2 h than in WT cells (Figures 6F, G). Moreover, the protein expression of Whi5-2×GFP in *atg1∆* cells was significantly lower than that in WT cells at 0, 1, and 4 h under starvation stimulation (Figures 6H, I). Thus, our results indicated that the protein but not mRNA expression levels of Rgd1 and Whi5 were influenced by *ATG1* during autophagy.

Next, we further explored whether Atg1 influences the sub-cellular localization of Rgd1 and Whi5 under nitrogen starvation conditions. These yeast cells were stained with FM 4–64, a commonly used dye to probe the yeast vacuolar membrane, and then transferred to SD-N medium for 0, 1, 2, 3 and 4 h, respectively. From the results, we observed that both Rgd1-2×GFP and Whi5-2×GFP were localized outside the vacuole in WT and *atg1∆* cells at all the 5 time points under starvation conditions (Supplementary Figures S3A–D), demonstrating that the protein degradation of Rgd1 or Whi5 might be independent of the autophagic process. Thus, our results indicated that Atg1 is not involved in altering the intracellular distribution of Rgd1 and Whi5 relative to vacuoles in autophagy.

### 3.7 Rgd1 and Whi5 potentially interacts with Atg1 to be involved in regulating autophagy

**FIGURE 7:**
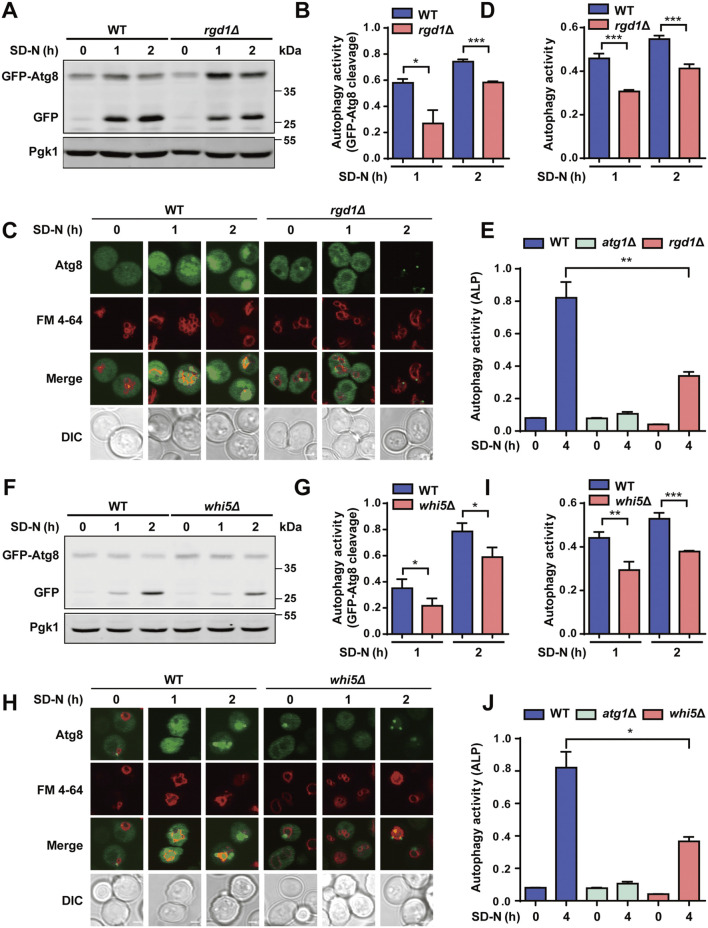
Rgd1 and Whi5 are crucial for regulating autophagy. **(A, B)** The cleavage of GFP-Atg8 was detected by immunoblotting in WT and *rgd1∆* yeast strains under nitrogen starvation for 1 and 2 h. The ratios between free GFP and the total amounts of free GFP and GFP-Atg8 were calculated for each yeast strain. **(C, D)** The GFP signals retained within vacuoles were observed using confocal microscopy in WT and *rgd1∆* strains expressing GFP-Atg8, which were stained with the dye FM 4–64 and cultured in SD-N medium for 0, 1 and 2 h. The images were analyzed to determine the yeast autophagy activity in WT and *rgd1∆* cells. Scale bar, 2 μm. **(E)** ALP activity was detected in WT and *rgd1∆* yeasts with nitrogen deprivation treatment for 0 and 4 h. The experimental procedures for **(F)** the GFP-Atg8 immunoblotting assay, **(G)** detection of the ratios between free GFP and the total amounts of free GFP and GFP-Atg8, **(H)** the GFP-Atg8 fluorescent assay, **(I)** measurement of autophagy activity using DeepPhagy, and **(J)** analyses of ALP activity conducted in WT and *whi5∆* yeast strains are shown. Scale bar, 2 μm. DIC, differential interference contrast. All experiments were performed independently three times. The two-sided t-test was used for statistical analyses. *p < 0.05; **p < 0.01; ***p < 0.001.

Here, we tested whether Rgd1 and Whi5 participate in modulating autophagy triggered by nitrogen starvation. The WT and *rgd1∆* cells were separately transformed with a plasmid expressing GFP-Atg8 and then treated with SD-N medium for 0, 1 and 2 h, respectively. The immunoblotting analyses indicated that the ratios between free GFP and the total amounts of free GFP and GFP-Atg8 were significantly lower in the *rgd1∆* strain than in the WT strain (Figures 7A, B). The accumulation of free GFP, generated from GFP-Atg8, within vacuoles in the WT and *rgd1∆* strains was observed via confocal fluorescence microscopy, and we found that the number of cells with free GFP molecules retained within vacuoles was greater among WT cells than among *rgd1∆* cells (Figure 7C). Then, we used DeepPhagy to quantitatively measure autophagy activity in WT and *rgd1∆* cells, respectively (Zhang et al., 2019). Our analyses indicated that the activity of autophagy was dramatically reduced in *rgd1∆* cells compared to WT cells (Figure 7D; Supplementary Figure S4A). Additionally, to evaluate the influence of Rgd1 on autophagy activity in yeast, a Pho8∆60 assay was employed as a quantitative readout (Klionsky et al., 2007; Klionsky et al., 2021). Alkaline phosphatase (ALP) activity was separately detected in WT cells (as the positive control), *atg1∆* cells (as the negative control) and *rgd1∆* cells after nitrogen starvation for 4 h. The analyses illustrated that the ALP activity in *rgd1∆* cells was much lower than that in WT cells (Figure 7E).

The same experimental procedure was performed to assess the potential regulatory role of Whi5 during yeast autophagy in response to starvation stimulation. Using the GFP-Atg8 immunoblotting assay, we found that the cleavage of GFP-Atg8 was significantly attenuated in yeast cells with deletion of *WHI5* compared to WT cells (Figures 7F, G). Also, we found that less free GFP was retained within vacuoles in *whi5∆* cells than in WT cells (Figure 7H). Consistent with this finding, the autophagy activity quantified by DeepPhagy was lower in *whi5∆* cells than in WT cells (Figures 7H, I; Supplementary Figure S4B). Additionally, by employing the Pho8∆60 assay, we found that ALP activity was lower in *whi5∆* cells than in WT cells upon nitrogen starvation stimulation (Figure 7J). In summary, our results supported that both Rgd1 and Whi5 might be critical for the autophagic process in *S. cerevisiae*.

**FIGURE 8:**
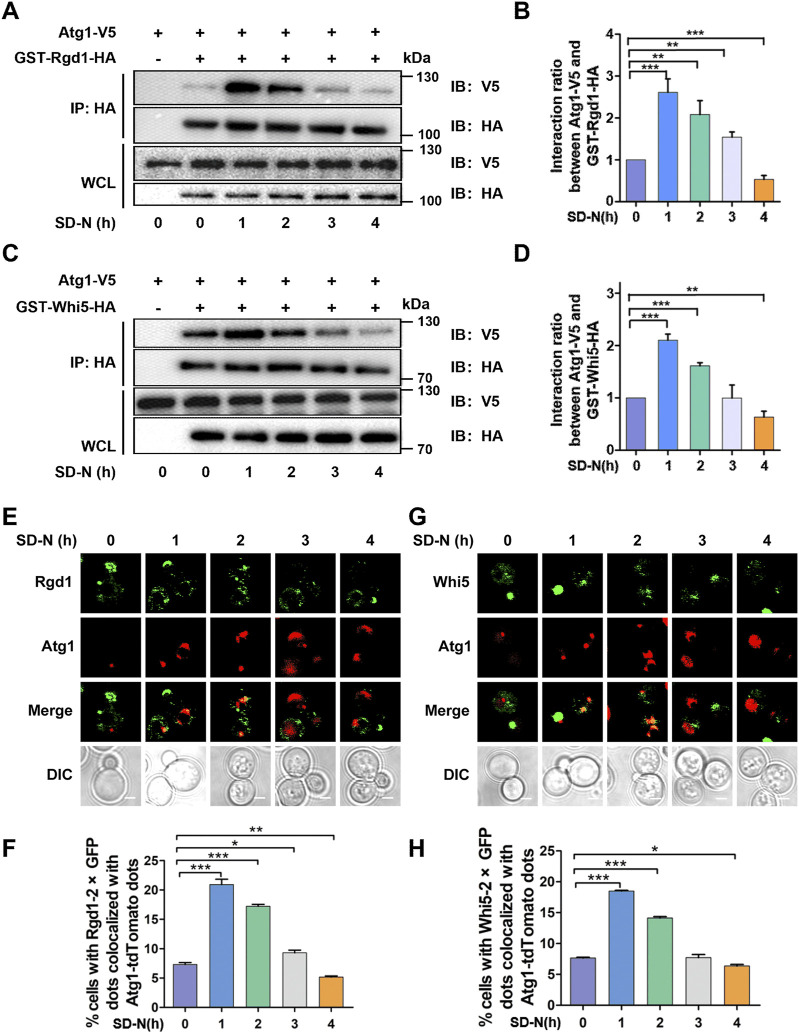
Atg1 interacts with Rgd1 and Whi5 during autophagic process. **(A)** The WT cells expressing V5-tagged Atg1 and HA-tagged Rgd1 were separately cultured in SD-N medium for 0, 1, 2, 3, and 4 h, respectively, and the yeasts were harvested and lysed. Then, the Co-IP assay was performed using anti-HA agarose beads, and the immunoprecipitates were detected through immunoblotting with anti-V5 and anti-HA antibodies. **(B)** The relative intensities of V5-tagged Atg1 were analyzed at indicated timepoints. **(C)** The Co-IP assay for analyzing the potential interaction between Atg1 and Whi5 during the autophagic process and **(D)** the quantitative measurement of relative expression of V5-tagged Atg1. **(E)** The WT cells expressing Rgd1-2×GFP and Atg1-tdTomato were cultured in SD-N medium for 0, 1, 2, 3, and 4 h, respectively, and then observed via confocal microscopy. Scale bar, 2 μM. **(F)** The proportion of cells with the colocalization between Rgd1-2×GFP and Atg1-tdTomato was calculated through analyzing >300 cells. **(G)** The confocal observation for Whi5, and **(H)** the proportion of cells with Whi5-2×GFP colocalized with Atg1-tdTomato was calculated through analyzing >300 cells. Scale bar, 2 μM. DIC, differential interference contrast. All experiments were repeated three times independently. The two-sided t-test was employed for statistical analyses. *p < 0.05; **p < 0.01; ***p < 0.001.

To validate whether the two proteins, Rgd1 and Whi5, physically interact with Atg1, we generated yeast cells expressing Atg1 with a FLAG tag and Rgd1 or Whi5 tagged with glutathione S-transferase (GST) and hemagglutinin (HA), respectively. Using the Co-IP assay, we observed that both Rgd1 and Whi5 potentially interact with Atg1 *in vivo* (Figures 8A–D). To further explore the potential interaction between Atg1 and Rgd1 or Whi5 during the autophagic process, the cells expressing V5-tagged Atg1 and HA-tagged Rgd1 or HA-tagged Whi5 were cultured in SD-N medium for 0, 1, 2, 3, and 4 h, respectively. From the results, it was found that the physical interaction between Atg1 and Rgd1 was markedly enhanced at 1 and 2 h, compared with that at 0 h (Figures 8A, B). This result suggested that the potential interaction of Atg1 and Rgd1 might be increased at the early stages of the autophagic process. Also, similar results were obtained for the potential interaction between Atg1 and Whi5 during autophagy (Figures 8C, D). Moreover, to investigate the sub-cellular colocalization of the two proteins and Atg1 in the process of autophagy, the WT cells expressing Rgd1 or Whi5 C-terminally tagged with 2×GFP as well as Atg1 tagged with an RFP variant (Atg1-tdTomato) were constructed. The yeasts were cultured and then treated by nitrogen starvation for 0, 1, 2, 3, and 4 h, respectively (Figures 8E–H). In our results, it was found that there existed a partial but clear colocalization between Atg1-tdTomato and Rgd1-2×GFP or Whi5-2×GFP (Figures 8E, G). In contrast to the time point of 0 h, the proportion of cells expressing Rgd1-2×GFP or Whi5-2×GFP colocalized with Atg1-tdTomato dots at 1 or 2 h was significantly enhanced (Figures 8F, H), supporting that the potential interaction between Atg1 and Rgd1 or Whi5 might be increased at early stages of autophagy. Taken together, our results demonstrated that Rgd1 and Whi5 potentially interact with Atg1, and are required for bulk autophagy.

### 3.8 Whi5 is phosphorylated by Atg1 to regulate Atg1 puncta formation during autophagy initiation

**FIGURE 9:**
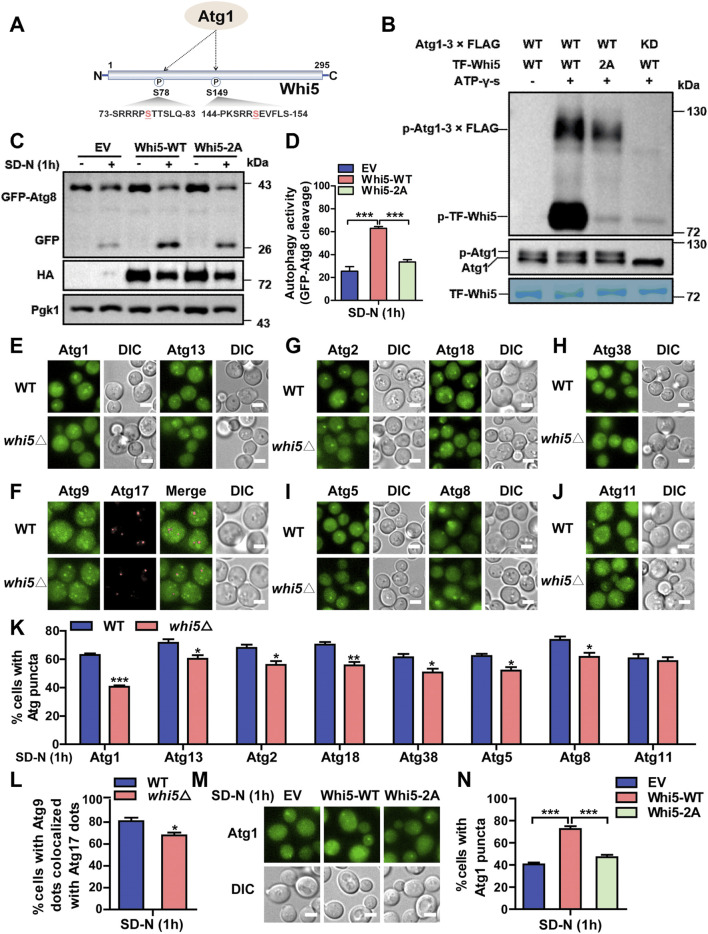
Whi5 is phosphorylated by Atg1 and involved in altering Atg1 puncta formation during autophagy initiation. **(A)** The potential p-sites on Whi5 protein modified by Atg1 were predicted by the GPS algorithm. **(B)** *In vitro* kinase assays were conducted. The purified WT Whi5 or Whi5-2A from *Escherichia coli* were separately incubated with purified SD-N medium-treated intact Atg1 or Atg1 KD from yeast cells, and the phosphorylation levels of Whi5 or its mutant were detected using anti-thioP antibody. **(C, D)** Immunoblotting analyses of the cleavage of GFP-Atg8 were conducted in *whi5∆* yeast cells separately expressing control plasmid, Whi5-WT or Whi5-2A that were treated with SD-N medium for 1 h. **(E)** WT and *whi5∆* yeast strains expressing Atg1-GFP or Atg13-2×GFP were cultured in SD-N medium for 1 h, and the image of Atg1 puncta were recorded using fluorescence microscopy. Scale bar, 3 μm. **(F)** The yeast cells expressing Atg17-2×mCherry and Atg9-2×GFP were treated using SD-N medium, and the images were observed and captured under fluorescence microscopy. Scale bar, 3 μm. **(G–J)** The yeast strains expressing Atg2-2×GFP, Atg18-2×GFP, Atg38-2×GFP, Atg5-2×GFP, GFP-Atg8 or Atg11-2×GFP were incubated under nitrogen starvation, and the images of Atg puncta were observed and captured. Scale bar, 3 μm. **(K)** The analyses of the proportion of cells expressing Atg puncta, including Atg1, Atg13, Atg2, Atg18, Atg38, Atg5, Atg8, and Atg11. *n* = 300 cells were pooled and measured from three independent experiments. **(L)** The quantification of the colocalization of Atg9 and Atg17 in WT and *whi5∆* cells from **(F)**. *N* = 300 cells were analyzed from three independent experiments. **(M, N)** The *whi5∆* yeasts expressing Atg1-GFP were transformed with a control plasmid, a plasmid expressing intact Whi5 and a plasmid expressing Whi5-2A, and then were cultured in SD-N medium for 1 h. The proportions of cells with Atg1 puncta were calculated. *n* = 300 cells were pooled from three independent experiments. Scale bar, 3 μm. DIC, differential interference contrast; EV, empty vector. All experiments were repeated three times independently. The two-sided t-test was employed for statistical analyses. *p < 0.05; **p < 0.01; ***p < 0.001.

Using Group-based Prediction System (GPS) (Xue et al., 2008; Wang et al., 2020), our previously developed algorithm for prediction of PK-specific p-sites, it was demonstrated that two serine residues of S78 and S149 in Whi5 might be potentially phosphorylated by Atg1 (Figure 9A). Then, we carefully checked the phosphoproteomic datasets to find whether the 2 p-sites were quantified in this study. From the results, we found that the pS78 site of Whi5 was quantifiable in WT cells at 1 and 4 h and in *atg1∆* cells at 4 h, while pS149 of Whi5 was detected only in WT cells at 1 h (Supplementary Figure S5). At 4 h, the intensity value of Whi5 pS78 in WT cells was higher than that in *atg1∆* cells (Supplementary Figure S5). Thus, our results suggested that the deletion of *ATG1* might decrease the phosphorylation of Whi5, at least at the pS78 site, in the late stage of autophagy. Next, yeast *WHI5* gene was cloned into the *E. coli* (*E. coli*) expression vector, and the Whi5 protein were purified and used as a potential substrate. We separately immunoprecipitated intact Atg1 or Atg1 KD (D211A) PKs from yeast cells, and then incubated with yeast Whi5 for conducting *in vitro* kinase assays. It was observed that WT Atg1, but not KD Atg1, was able to phosphorylate Whi5 (Figure 9B), indicating Whi5 phosphorylation was in an Atg1 kinase activity-dependent manner. Furthermore, we used a site-directed mutagenesis method to convert serine into alanine, and consequently construct a WHI5 mutant with both S78A and S149A (Whi5-2A). In *in vitro* kinase assays, we found that the phosphorylation level of *WHI5* mutant was abolished (Figure 9B), demonstrating that the 2 p-sites of Whi5 might be truly modified by Atg1. To explore whether the 2 p-sites in Whi5 play a role in autophagy induced by nitrogen starvation, the *whi5∆* yeast cells expressing GFP-Atg8 were separately transformed with a control plasmid, a plasmid expressing intact Whi5, and a plasmid expressing the Whi5-2A mutant, and then cultured in SD-N medium for 1 h. Using the immunoblotting assay, we observed that expression of the intact Whi5 significantly increased autophagy activity, whereas expression of the Whi5 mutant had little influence on autophagy (Figures 9C, D). These results suggested that the two serine residues on Whi5 might be essential for Whi5 that functions in the autophagic process.

Next, we further explored the potential role of Whi5 protein in autophagy. Here, the fluorescent protein GFP fused with various Atg proteins, including Atg1, Atg13, Atg9, Atg2, Atg18, Atg38, Atg5, Atg8 and Atg11, were expressed in WT and *whi5∆* yeasts (Figures 9E–J), and the formation of Atg fluorescent puncta and subcellular localization of Atg9 were analyzed in yeast cells upon the treatment of nitrogen starvation (Figures 9K, L). From our results, it was observed that the knockout of *WHI5* significantly repressed the formation of Atg1 puncta under nitrogen deprivation conditions (Figure 9E). For Atg13 protein, a core component of Atg1 complex for autophagy initiation (Farré and Subramani, 2016; Wen and Klionsky, 2016), we found that the formation of Atg13 puncta was also repressed in the *whi5∆* strains, compared to that in WT cells (Figure 9E). Previously, it was reported that the recruitment of Atg9 vesicles to the phagophore assembly site (PAS) is essential during the process of nucleation (Farré and Subramani, 2016; Wen and Klionsky, 2016), and the PAS recruitment of Atg9 is mediated by interaction with HORMA domain of Atg13 at the early stage of autophagy (Suzuki et al., 2015). Thus, we used Atg17 as a PAS marker, and revealed that the deletion of *WHI5* reduced the co-localization of Atg9 and Atg17, demonstrating that Whi5 involved in influencing subcellular localization of Atg9 to PAS (Figure 9F). Meanwhile, Atg9 recruits the Atg2-Atg18 complex, which is important for the elongation of phagophore membrane (Suzuki et al., 2015). Consistently, we observed that the number of Atg2 and Atg18 puncta were reduced after the silence of *WHI5* in response to nitrogen starvation (Figure 9G). In addition, previous studies reported that phosphatidylinositol 3-kinase complex I (PI3KCI) associates with the PAS scaffold Atg1 complex through the interaction between Atg1 and Atg38 (Hitomi et al., 2023). Our results showed that knocking out *WHI5* decreased the formation of Atg38 puncta (Figure 9H). Moreover, Atg5 and Atg8 serve as key proteins in two ubiquitin-like systems, which contribute to phagophore expansion (Wen and Klionsky, 2016). The previous reports revealed that Atg1 interacts with Atg8 to promote autophagosome formation (Kraft et al., 2012), and the PAS recruitment of Atg12-Atg5-Atg16 is regulated via the association of Atg12 and Atg1 complex (Harada et al., 2019). In our analyses, it was demonstrated that the formation of Atg8 puncta as well as Atg5 puncta were suppressed in *whi5∆* yeasts, in contrast to WT cells (Figure 9I). Furthermore, we measured the effect of Whi5 on Atg11, an adaptor protein for selective autophagy (Yao et al., 2023b), and it was found that *WHI5* knockout did not alter the formation of Atg11 puncta (Figure 9J), suggesting that Whi5 might not be involved in selective autophagy. According to our findings, it was indicated that Whi5 participates in altering Atg1 puncta formation during autophagy initiation, which consequently results in influencing the puncta formation of other Atg proteins at the stages of nucleation and expansion.

Next, we further investigated whether the phosphorylation of Whi5 is involved in modulating Atg1 puncta formation during the autophagic process induced by nitrogen deprivation. Here, an empty plasmid, a plasmid expressing wild-type Whi5, and a plasmid expressing Whi5-2A mutant were individually transformed into the *WHI5* deletion cells expressing Atg1-GFP, and then the yeasts were treated with SD-N medium for 1 h. In our results, it was observed that, in comparison with the empty plasmid, rescuing the intact Whi5 significantly increased the number of Atg1 puncta (Figures 9M, N). In contrast with WT Whi5, the mutant of Whi5-2A significantly impaired the formation of Atg1 puncta (Figures 9M, N), demonstrating that Whi5 phosphorylation might be requited for Atg1 puncta formation during autophagic process. Taken together, our analyses indicated that the Whi5 is directly phosphorylated by the Atg1 kinase, and the phosphorylation of Whi5 is involved in modulating the formation of Atg1 puncta during autophagy initiation.

## 4 Discussion

Autophagy is a degradative and recycling process highly conserved from yeast to mammals that ensures the intracellular homeostasis through degradation of defective organelles or proteins (Ohsumi, 2014). Atg1 is an important member of the core autophagy machinery in *S*. *cerevisiae*, and plays an essential role in various steps of the autophagic process in response to nutrient deprivation, especially autophagosome formation (Ohsumi, 2014). During the initiation of autophagy, Atg1 acts as a scaffold protein to interact with Atg13 and Atg17 to facilitate the assembly of the Atg1 complex, eventually prompting autophagy activity (Kabeya et al., 2005; Cheong et al., 2008; Lin et al., 2018). Moreover, Atg1 is associated with Atg8 and delivered with autophagosomes to vacuoles, which leads to downregulation of the activity of the Atg1 complex (Kraft et al., 2012). The kinase activity of Atg1 is also crucial for sustaining autophagy activity. Several important substrates regulated by Atg1, including Atg9, Atg4 and Atg29 (Papinski et al., 2014; Sanchez-Wandelmer et al., 2017; Hu et al., 2019), have been identified to be involved in regulating the diverse stages of autophagy (Mizushima, 2010; Wang and Kundu, 2017). It has remained unclear how the nonkinase function and kinase activity of Atg1 synergistically modulate autophagy.

In this study, by integrating multi-omic datasets and the protein sequence features, a new AI framework, iAMD, was developed to computationally predict potentially new autophagy regulators from the Atg1 interactome. Using iAMD, we predicted 12 Atg1-interacting partners and 17 substrates as candidate autophagy regulators. Through a functional screening of 25 candidate genes with KO mutant strains, Rgd1 and Whi5 were validated to be potentially positive autophagy regulators, together with following experiments. According to our findings and the available knowledge, a signal web was modeled to highlight the important role of Atg1 in multiple steps of the autophagic process. Via integration of PPIs and multi-omic data, 67 Atg1-interacting proteins, including 65 known Atg proteins and autophagy regulators as well as two newly identified autophagy regulators, Rgd1 and Whi5, were presented in the Atg1-centered network during the process of autophagy.

**FIGURE 10:**
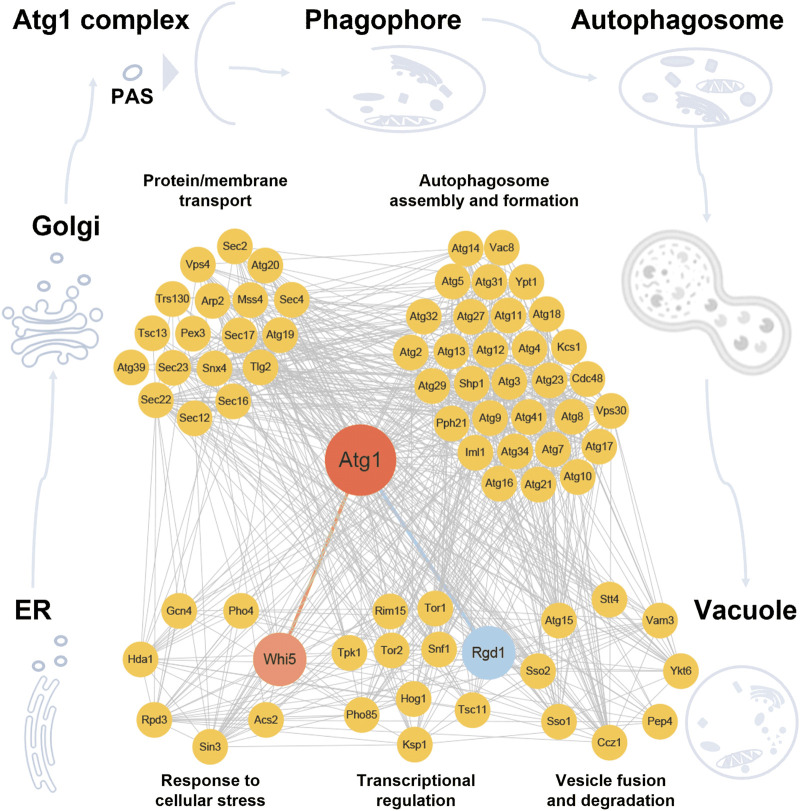
Computational working model of the Atg1-centered regulatory network. Based on previous knowledge and our findings of this study, the 75 experimentally identified Atg proteins and autophagy regulators were classified into five groups, including response to cellular stress, transcriptional regulation, protein/membrane transport, autophagosome assembly and formation, vesicle fusion and degradation, according to their biological roles during the process of autophagy. Yeast known PPIs were integrated from nine public databases. The Atg1-centered regulatory network was constructed and visualized with Cytoscape (Shannon et al., 2003).

According to their known functions during autophagy, the 75 proteins were classified into five groups, including response to cellular stress, transcriptional regulation, protein/membrane transport, autophagosome assembly and formation, and vesicle fusion and degradation (Figure 10). Previously, it was reported that *RGD1* was identified to be functional under various environmental stress, such as low potential of hydrogen (pH), heat shock and oxidative shock (Gatti et al., 2005). Thus, Rgd1 may induce autophagy in response to environmental stimuli. Also, the sucrose nonfermenting 1 (Snf1), the mammalian AMP-activated protein kinase (AMPK) homolog, has been characterized as a cellular energy sensor in yeasts, and is essential for autophagy activation through recruiting Atg1 during the glucose starvation (Wang et al., 2001; Papinski and Kraft, 2016; Yi et al., 2017; Mizuno et al., 2020). Moreover, the cyclin-dependent kinase Pho85 plays a critical role in sensing the alterations of nutrient restriction and salinity (Nishizawa et al., 2010), as well as autophagy regulation (Yang et al., 2010). Under multiple stimuli, such as energy and amino acid deprivations, the activity of TORC1 is inhibited, which eventually results in the activation of Atg1 complex for autophagy induction (Kamada et al., 2010; Yang et al., 2010; Jewell et al., 2013). Whi5 participates in transcriptional regulation through inhibiting SBF-dependent transcription, and its functional homolog is the retinoblastoma-associated protein RB1 in mammalian cells (Takahata et al., 2009; Schmoller et al., 2015; Qu et al., 2019; Barber et al., 2020). Previous studies have indicated that RB1 plays an essential role in regulating autophagy in mammals including humans (Jiang et al., 2010; Ruan et al., 2020). Interestingly, Whi5 was found to be required for bulk autophagy, and the p-sites of pS78 and pS149 in Whi5 were critical for sustaining autophagy activity induced by nitrogen starvation, and the phosphorylation of Whi5 involves in influencing the Atg1 puncta formation at the stage of autophagy. Previously, it was reported that Gcn4, a master transcription factor, is involved in autophagy modulation through altering the gene expression of *ATG1* (Huang et al., 2022). Sin3 and Rpd3, components of the histone deacetylase (HDAC) complex, were found to be necessary for modulating the expression of *ATG8* at the transcriptional level (Bartholomew et al., 2012). Also, it has been reported that the inhibition of an additional HDAC, Hda1, is able to promote autophagy activity (Robert et al., 2011). In conclusion, the modeled Atg1-centered network suggested that Atg1 might cooperate with its interacting partners as well as substrates to synergistically regulate each stage of the autophagic process.

Despite the overall effectiveness of iAMD framework, it still has certain limitations. The regulatory role of Atg1 in autophagy was modulated by several upstream kinases, including PKA, TORC1, and Snf1, as well as Atg1 autophosphorylation (Licheva et al., 2022). Autophosphorylation at T226 and S230 had been shown to directly affect Atg1 kinase activity and autophagy induction (Kijanska et al., 2010). Furthermore, PKA-mediated phosphorylation at S508 and S515 might influence Atg1 function by modulating PAS localization (Budovskaya et al., 2005). TORC1 directly regulates autophagy by targeting the Atg1 complex, and reciprocally, Atg1 modulates TORC1 signaling through both direct and indirect mechanisms (Hu et al., 2019). Similarly, Snf1 promoted autophagy through phosphorylation of Mec1 on the mitochondrial surface in an Atg1-dependent manner, and participates in a feedback loop with TORC1 (Yi et al., 2017; Hu et al., 2019). However, iAMD is not designed to systematically identify such upstream regulators, particularly those lacking reciprocal signaling relationships. Furthermore, the iAMD framework is currently limited in its ability to infer the time sequence of regulatory events within the Atg1-centered signaling network. As a result, the dynamic sequence of regulators in different stages of autophagy cannot be determined based on model predictions alone. This limitation underscores the importance of experimental validation to functionally interpret the computational results generated by iAMD.

Next, we evaluated the extensibility of iAMD, by analyzing additional 6 PKs also involved in regulating autophagy, including TORC1, Rim15, Yak1, Gcn2, Slt2, and Npr1 (Oliveira et al., 2015; Dokladal et al., 2021a; Dokladal et al., 2021b). For the 6 PKs, only phosphoproteomic datasets were available, and their interacting partners were obtained from the 9 public PPI databases (Xenarios et al., 2002; Razick et al., 2008; Das and Yu, 2012; Kerrien et al., 2012; Licata et al., 2012; Calderone et al., 2013; Chatr-Aryamontri et al., 2017; Kotlyar et al., 2019; Szklarczyk et al., 2021). For prediction of PK-interacting partners also involved in regulating autophagy, the AUC values of different models ranged from 0.779 to 0.850 (Supplementary Figures S6A–F). For prediction of PK substrates involved in autophagy, the AUC values of different models ranged from 0.814 to 0.894 (Supplementary Figures S6G–L). By comparison, iAMD exhibited a superior performance than other method, and our results demonstrated iAMD to be a highly transferrable method. Taken together, this work not only provided a powerful AI framework for analyzing multi-omic data, but also revealed new autophagy players in the context of important PKs.

## Data Availability

The datasets presented in this study can be found in online repositories. The names of the repository/repositories and accession number(s) can be found in the article/Supplementary Material.
