# Supplementary material for: Omic AI reveals new autophagy regulators from the Atg1 interactome in Saccharomyces cerevisiae
Source: Front Cell Dev Biol. 2025 Apr 29;13:1554958. doi: 10.3389/fcell.2025.1554958 (PMC12069372; doi:10.3389/fcell.2025.1554958)
Supplement: Supplementary file 1 [file DataSheet1.zip › Data Sheet 1/Supplementary_Material.docx]

Supplementary Material

## Supplementary Figures


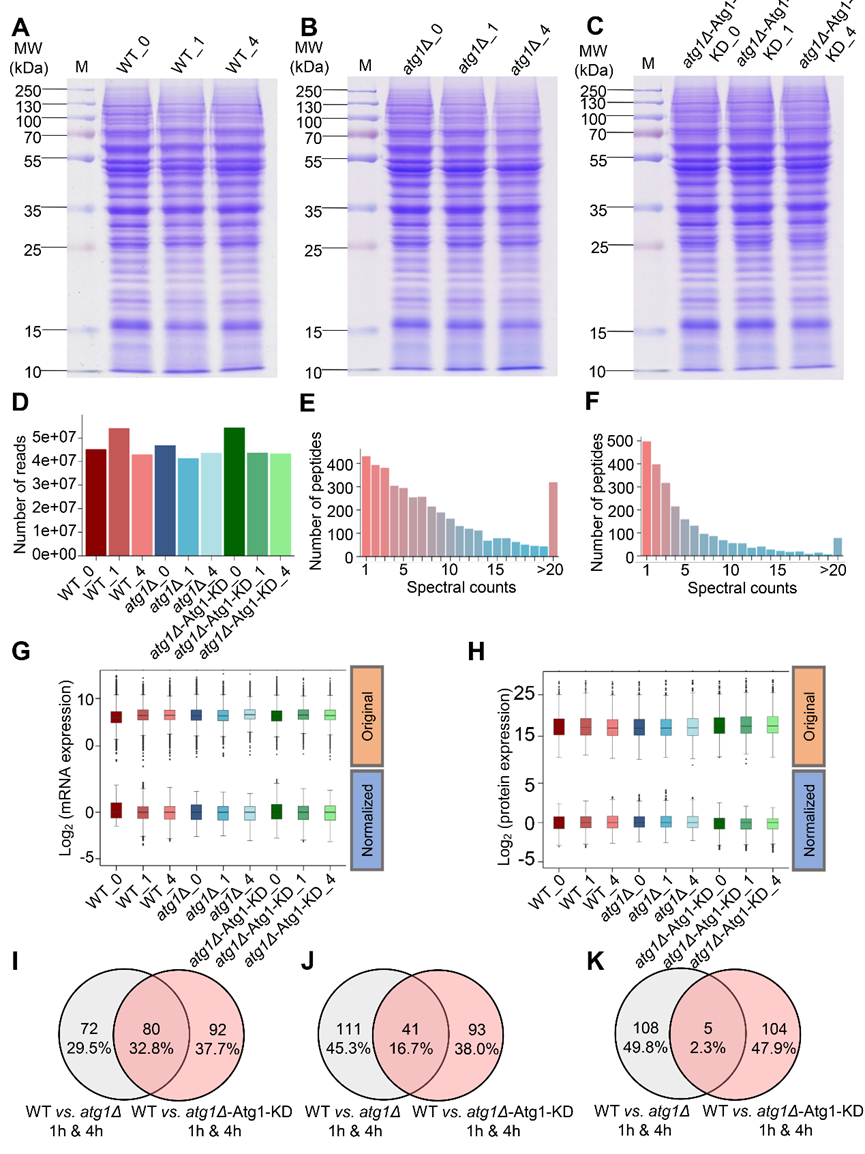


**Supplementary Figure 1.** Profiling of the multiomics datasets for autophagy. (**A**, **B** and **C**) WT, *atg1∆* and *atg1∆*-Atg1 KD yeast samples at each time point were examined by 12% SDS–PAGE. (**D**) Numbers of reads detected in WT, *atg1∆* and *atg1∆*-Atg1 KD yeast cells. (**E**) Distribution of the numbers of peptides in mappable proteins. (**F**) Distribution of the numbers of peptides in mappable phosphoproteins. (**G**) Distribution of the FPKM values of mRNAs with or without normalization. (**H**) Distribution of the intensity values of proteins with or without normalization. (**I**, **J** and **K**) In total, 244 genes, 245 proteins and 217 phosphoproteins were potentially regulated by *ATG1* in the autophagic process.


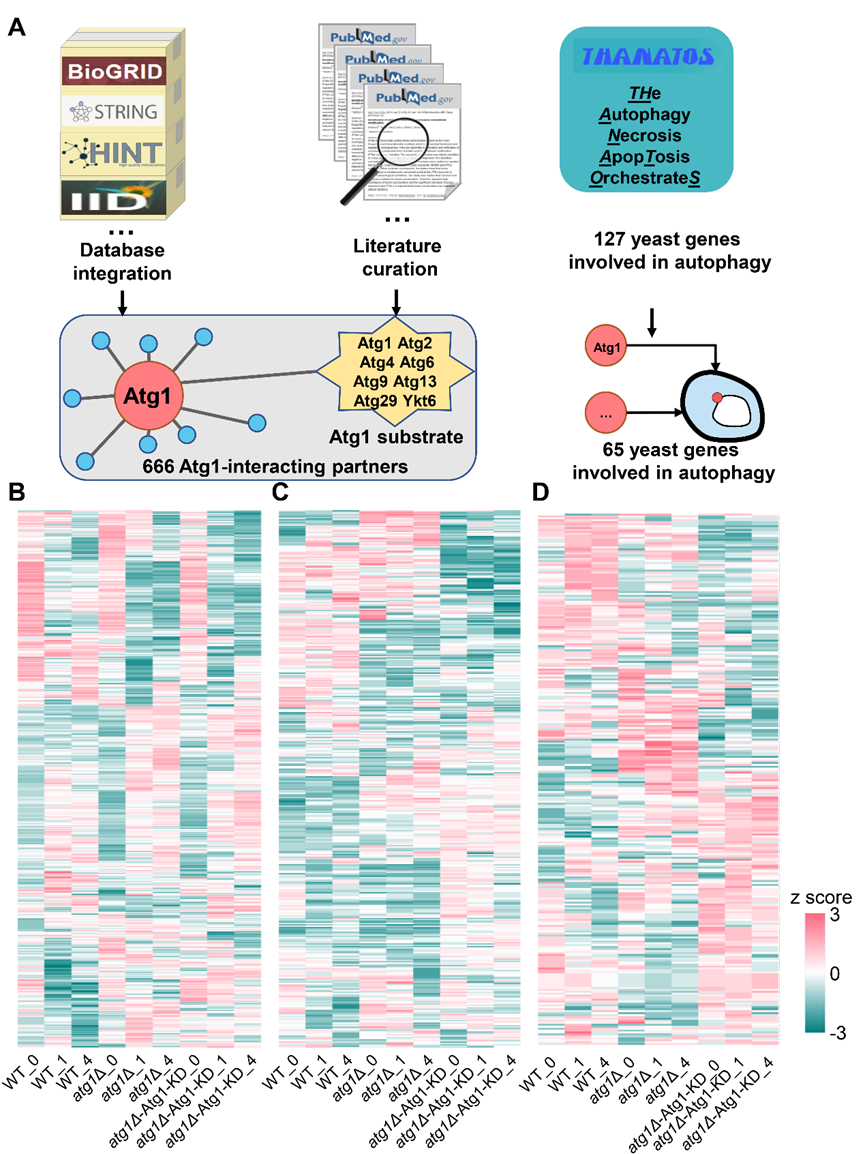


**Supplementary Figure 2.** Atg1-interacting partners collection and profiling of expression level in 3 yeast strains treated with SD-N medium for 0, 1 and 4 h. (**A**) Data collection of Atg1-interacting partners, substrates and yeast genes involved in autophagy. (**B**) mRNA expression levels of 622 Atg1-interacting partners after normalization. (**C**) Normalized protein expression levels of 421 proteins that interact with Atg1. (**D**) Expression levels of 274 Atg1-interacting phosphoproteins.


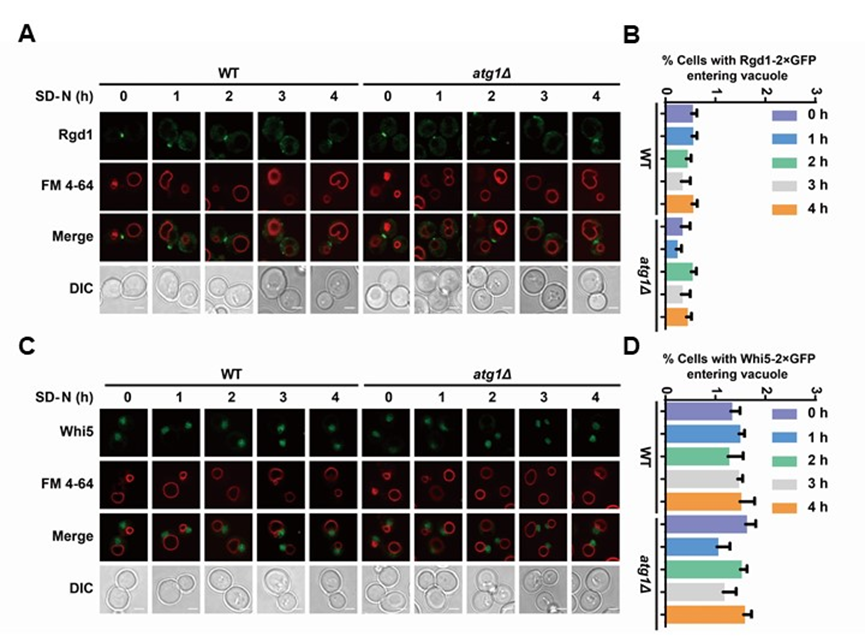


**Supplementary Figure 3.** The localization of Rgd1-2×GFP or Whi5-2×GFP relative to vacuoles in WT and *atg1∆* yeasts during autophagy. (**A and B**) WT and *atg1∆* yeasts expressing Rgd1-2×GFP were stained with the dye FM4-64, cultured with SD-N medium, and observed using confocal microscopy. The proportions of cells expressing Rgd1-2×GFP with GFP signals accumulated in vacuoles were calculated among > 300 cells for each strain. Scale bar, 2 μM. (**C and D**) The distribution of Whi5-2×GFP relative to vacuoles in WT and *atg1∆* yeasts. The proportions of cells expressing Whi5-2×GFP with GFP molecules retained within vacuoles were counted in > 300 cells for each strain. Scale bar, 2 μM. DIC, differential interference contrast. All experiments were independently repeated 3 times. Statistical analyses were conducted using the two-sided t test.


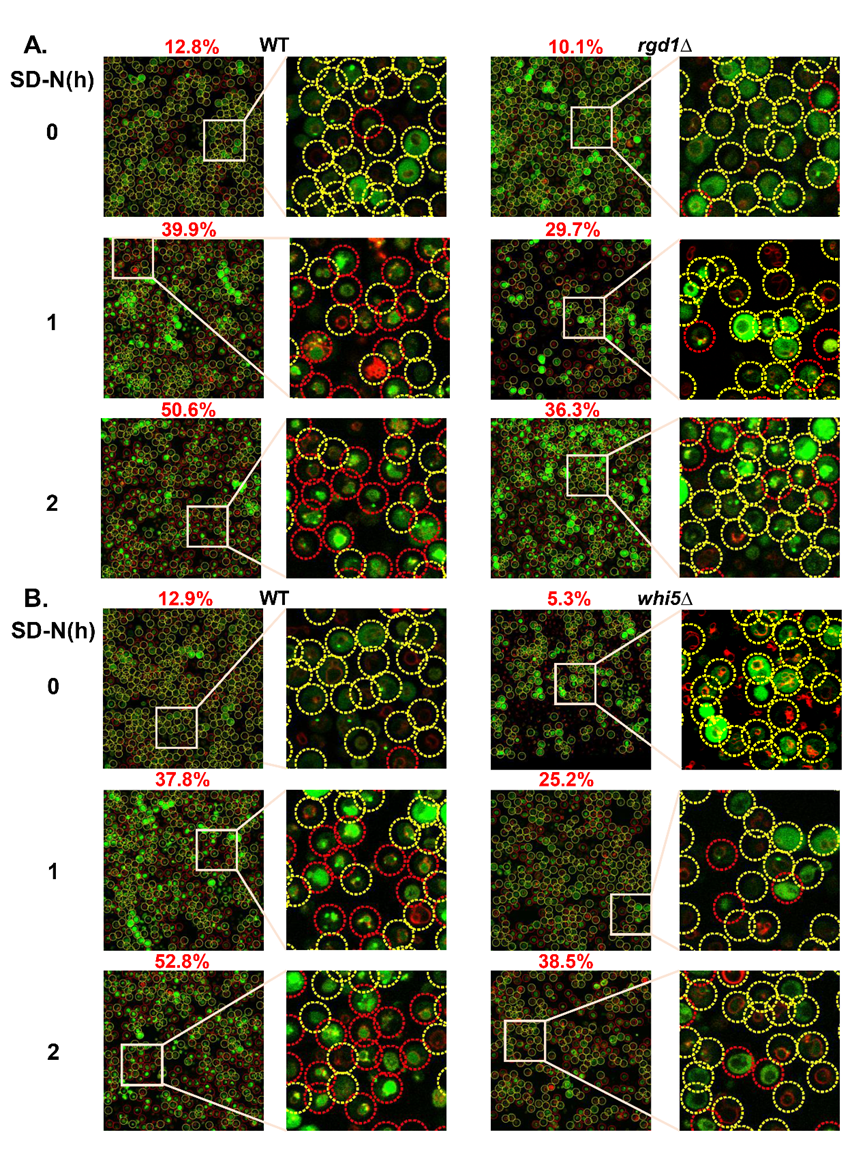


**Supplementary Figure 4.** Quantification of autophagy activity in WT, *rgd1∆*, and *whi5∆* cells by DeepPhagy. (**A**) Quantification of autophagy activity in WT and *rgd1∆* cells. (**B**) Quantification of autophagy activity in WT and *whi5∆* cells.


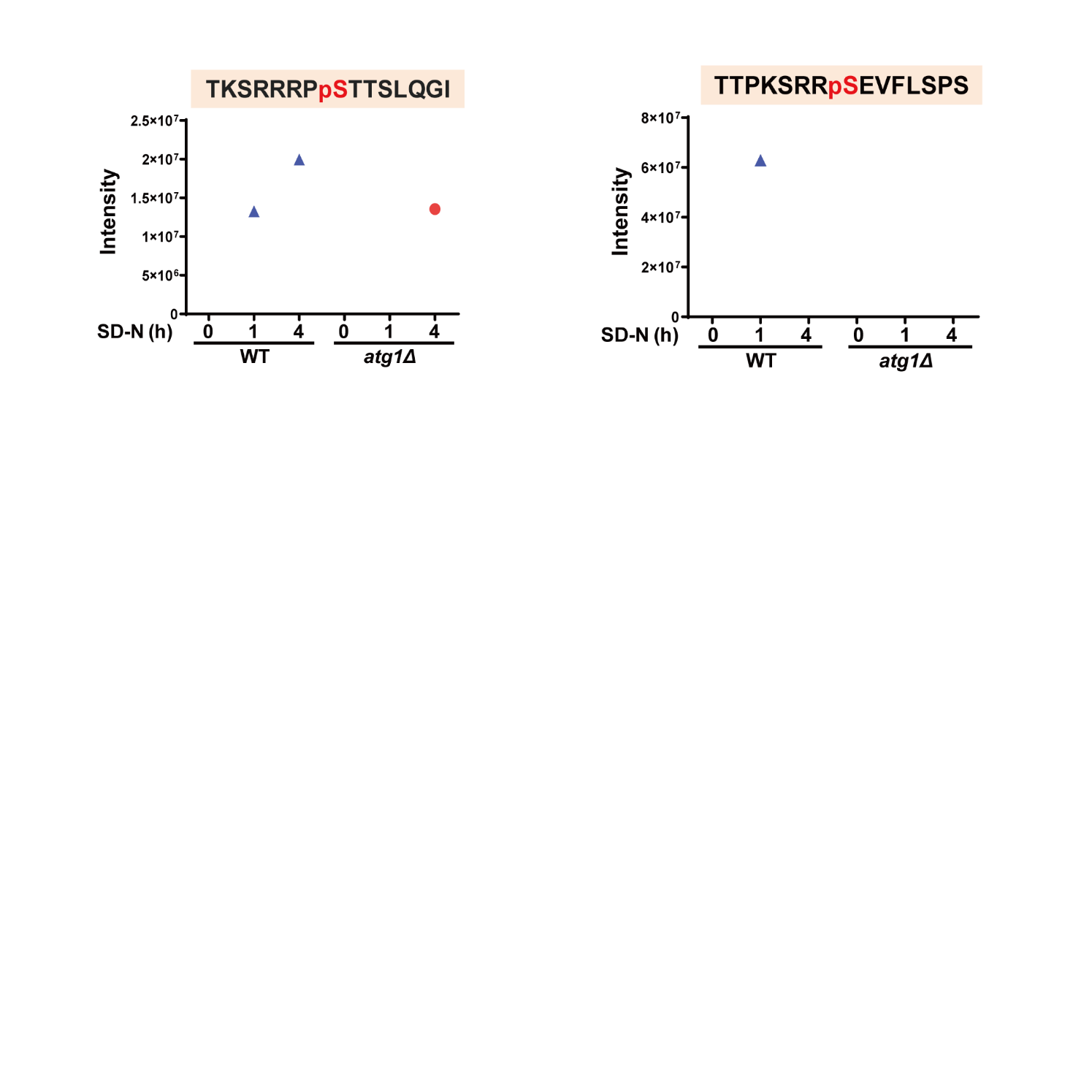


**Supplementary Figure 5.** Whi5 phosphorylation influenced by Atg1 during autophagic process. Intensity of p-sites on serine residues at the 78th and 149th sites of Whi5 from phosphoproteomic data.


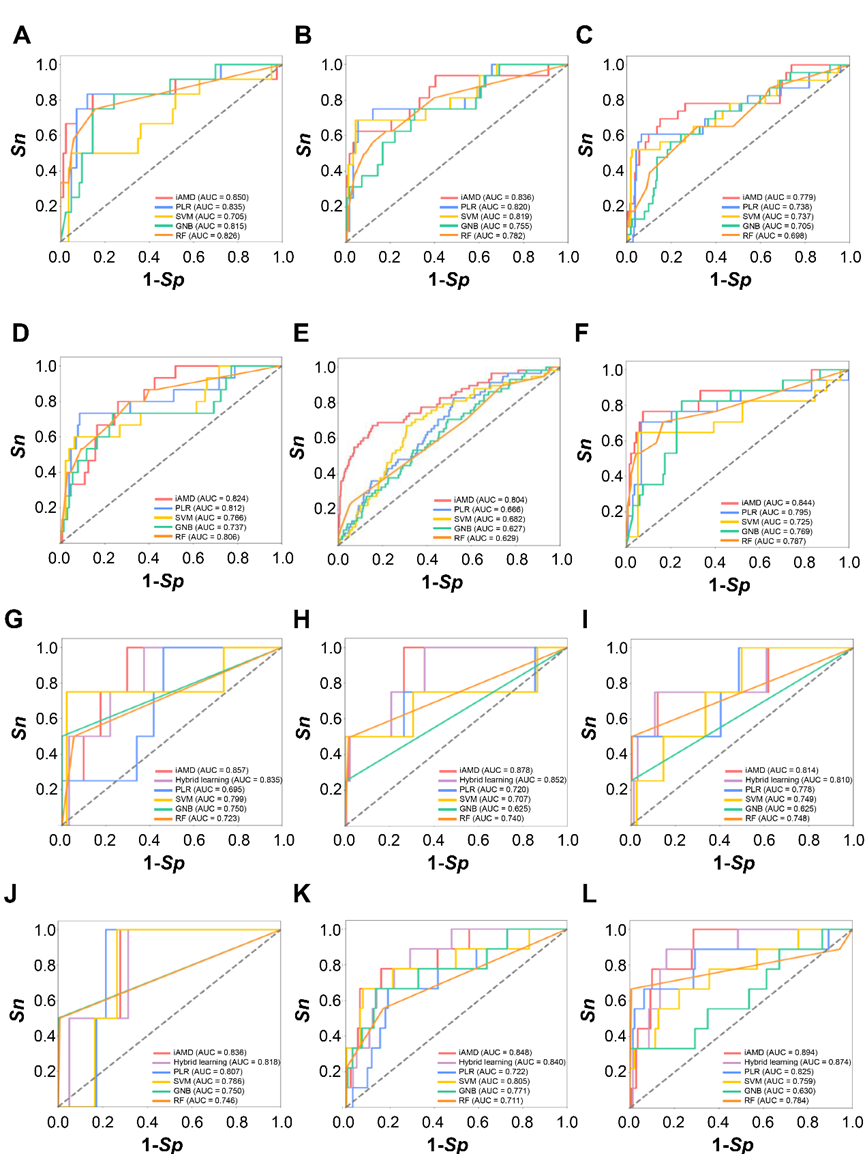


**Supplementary Figure 6.** Performance and comparison of iAMD and other machine learning methods for the prediction of autophagy regulators from the interactomes of other PKs involved in regulating autophagy. (**A**) AUC values of the TORC1-interacting partner predictive model using iAMD, PLR, SVM, GNB, and RF. (**B-F**) AUC values of predictive models for inferring the interacting partner of (**B**) Gcn2, (**C**) Rim15, (**D**) Yak1, (**E**) Slt2 and (**F**) Npr1, by using the methods of iAMD, PLR, SVM, GNB, and RF. (**G**) AUC values of computational predictor for inferring the TORC1 substrate using iAMD, a hybrid learning method without meta-learning, PLR, SVM, GNB, and RF. (**H-L**) AUC values of predictive model for predicting the substrate of (**H**) Gcn2, (**I**) Rim15, (**J**) Yak1, (**K**) Slt2 and (**L**) Npr1, by using iAMD, a hybrid learning method without meta-learning, PLR, SVM, GNB, as well as RF.
